# Genome-wide CRISPR/Cas9 knockout screening uncovers a novel inflammatory pathway critical for resistance to arginine-deprivation therapy

**DOI:** 10.7150/thno.51795

**Published:** 2021-01-25

**Authors:** Cheng-Ying Chu, Yi-Ching Lee, Cheng-Han Hsieh, Chi-Tai Yeh, Tsu-Yi Chao, Po-Hung Chen, I-Hsuan Lin, Tsung-Han Hsieh, Jing-Wen Shih, Chia-Hsiung Cheng, Che-Chang Chang, Ping-Sheng Lin, Yuan-Li Huang, Tsung-Ming Chen, Yun Yen, David K. Ann, Hsing-Jien Kung

**Affiliations:** 1 TMU Research Center of Cancer Translational Medicine, Taipei Medical University, Taipei 110, Taiwan.; 2 CRISPR Gene Targeting Core Lab, Taipei Medical University, Taipei 110, Taiwan.; 3 Department of Medical Research & Education, Taipei Medical University - Shuang Ho Hospital, New Taipei City.; 4 Department of Hematology & Oncology, Taipei Medical University - Shuang Ho Hospital, New Taipei City, Taiwan.; 5 Graduate Institute of Clinical Medicine, College of Medicine, Taipei Medical University, Taipei City, Taiwan.; 6 Institute of Biological Chemistry, Academia Sinica, Taipei, Taiwan.; 7 Bioinformatics Core Facility, The University of Manchester, Manchester, United Kingdom.; 8 Joint Biobank, Office of Human Research, Taipei Medical University, Taipei, Taiwan.; 9 Ph.D. Program for Cancer Biology and Drug Discovery, College of Medical Science and Technology, Taipei Medical University, Taipei 110, Taiwan.; 10 Graduate Institute of Cancer Biology and Drug Discovery, College of Medical Science and Technology, Taipei Medical University, Taipei 110, Taiwan.; 11 Department of Biochemistry and Molecular Cell Biology, School of Medicine, College of Medicine, Taipei Medical University, Taipei, Taiwan.; 12 Graduate Institute of Medical Sciences, College of Medicine, Taipei Medical University, Taipei, Taiwan.; 13 The Ph.D. Program for Translational Medicine, College of Medical Science and Technology, Taipei Medical University, Taipei 110, Taiwan.; 14 Department of Medical Laboratory Science and Biotechnology, Taipei Medical University, Taipei 110, Taiwan.; 15 Department of Biotechnology, College of Medical and Health Science, Asia University, Taichung, Taiwan.; 16 Department of Medical Research, China Medical University Hospital, China Medical University, Taichung, Taiwan.; 17 Department and Graduate Institute of Aquaculture, National Kaohsiung University of Science and Technology, Kaohsiung 81157, Taiwan.; 18 Department of Diabetes and Metabolic Diseases Research, Irell & Manella Graduate School of Biological Sciences, Beckman Research Institute, City of Hope, Duarte, CA, United States.; 19 Institute of Molecular and Genomic Medicine, National Health Research Institutes, Zhunan, Miaoli County 350, Taiwan.; 20 Department of Biochemistry and Molecular Medicine, Comprehensive Cancer Center, University of California at Davis, Sacramento, CA 95817, United States.

**Keywords:** arginine starvation, ADI resistance, CRISPR/Cas9, TREM1, CCL2

## Abstract

Arginine synthesis deficiency due to the suppressed expression of ASS1 (argininosuccinate synthetase 1) represents one of the most frequently occurring metabolic defects of tumor cells. Arginine-deprivation therapy has gained increasing attention in recent years. One challenge of ADI-PEG20 (pegylated ADI) therapy is the development of drug resistance caused by restoration of ASS1 expression and other factors. The goal of this work is to identify novel factors conferring therapy resistance.

**Methods:** Multiple, independently derived ADI-resistant clones including derivatives of breast (MDA-MB-231 and BT-549) and prostate (PC3, CWR22Rv1, and DU145) cancer cells were developed. RNA-seq and RT-PCR were used to identify genes upregulated in the resistant clones. Unbiased genome-wide CRISPR/Cas9 knockout screening was used to identify genes whose absence confers sensitivity to these cells. shRNA and CRISPR/Cas9 knockout as well as overexpression approaches were used to validate the functions of the resistant genes both *in vitro* and in xenograft models. The signal pathways were verified by western blotting and cytokine release.

**Results:** Based on unbiased CRISPR/Cas9 knockout screening and RNA-seq analyses of independently derived ADI-resistant (ADIR) clones, aberrant activation of the TREM1/CCL2 axis in addition to ASS1 expression was consistently identified as the resistant factors. Unlike ADIR, MDA-MB-231 overexpressing ASS1 cells achieved only moderate ADI resistance both *in vitro* and *in vivo*, and overexpression of ASS1 alone does not activate the TREM1/CCL2 axis. These data suggested that upregulation of TREM1 is an independent factor in the development of strong resistance, which is accompanied by activation of the AKT/mTOR/STAT3/CCL2 pathway and contributes to cell survival and overcoming the tumor suppressive effects of ASS1 overexpression. Importantly, knockdown of TREM1 or CCL2 significantly sensitized ADIR toward ADI. Similar results were obtained in BT-549 breast cancer cell line as well as castration-resistant prostate cancer cells. The present study sheds light on the detailed mechanisms of resistance to arginine-deprivation therapy and uncovers novel targets to overcome resistance.

**Conclusion:** We uncovered TREM1/CCL2 activation, in addition to restored ASS1 expression, as a key pathway involved in full ADI-resistance in breast and prostate cancer models.

## Introduction

It is now well recognized that tumor development requires metabolic adaptation to cope with rapid cell division as well as the hypoxic and nutritional-deprived tumor microenvironment 1. This metabolic reprogramming, however, also exposes tumor cells' vulnerabilities for special nutritional requirements, which can be exploited therapeutically. One of the most frequently observed metabolic deficiencies of tumor cells is the diminished ability to synthesize arginine due to the suppressed expression of ASS1 (argininosuccinate synthetase 1), rendering these cells arginine-auxotrophic or dependent on exogenous arginine for survival 2. The tumor-specific suppression of ASS1 is thought to divert aspartate, the precursor for intracellular arginine, for nucleotide synthesis needed for rapid cell growth 3. Targeting exogenous arginine by arginine-metabolizing enzymes such as arginase, arginine decarboxylase and arginine deiminase (ADI) has gained increasing attention as therapies to treat a variety of cancers 4. ADI-PEG20 (pegylated ADI) has undergone at least 20 phase I/II clinical trials with excellent safety profiles 5, 6. ADI-PEG20 has demonstrated encouraging anti-tumor activities in hepatocellular carcinoma in phase II trials 7, but as a monotherapy, did not meet its targeted goal in a phase III trial 8. More recent trials have shown medical benefits in combination with other agents in a number of cancers 9-12. Indeed, co-targeting of ADI-PEG20 with several clinically approved drugs including HDAC inhibitor Vorinostat significantly increased the efficacy 13.

One of the challenges in ADI-based therapy is the development of intrinsic resistance by the upregulation of ASS1 in treated cancer cells 14. Several mechanisms have been proposed, including demethylation of the ASS1 promoter 15, 16 and the activation of Myc oncogene which drives the expression of ASS1 17, 18. It is likely that depending on the types of cancers and the tumor microenvironment, different means have evolved to confer ADI resistance. While restoration of ASS1 expression seems to be necessary to provide the much-needed arginine to maintain metabolic balance during therapy, there may be other contributors to ADI resistance. For instance, in some of the ADI-resistant cells, the activation of Myc not only upregulates ASS1 expression but also contributes to other pathways critical for resistance. In melanoma cells, Myc also upregulates glycolysis and glutaminolysis independent of ASS1 overexpression 17. Enhanced AXL and EPH2 tyrosine kinase activities and the activation of down-stream PI3K/AKT signaling are also observed in resistant cells 19. Activation of the PI3K/AKT pathway is important to offset the tumor-suppressing effect of ASS1, known to be a suppressor of AKT activation 20. Likewise, in sarcoma cells, Myc is also elevated and stabilized to contribute to metabolic reprogramming and susceptibility to other drugs 21. By contrast, in lung cancer, Myc overexpression confers sensitivity, rather than resistance, to ADI treatment 22. These studies suggest that there are cellular factors beyond ASS1 that critically contribute to ADI resistance and are likely to be cancer or cell type specific. As direct targeting ASS1 may affect the metabolism of normal cells, these resistant factors and their associated pathways offer additional opportunities as targets to overcome ADI resistance.

Previously, we have used ASS1-low breast (e.g., MDA-MB-231) 23, 24 and prostate cancer (e.g., CWR22Rv1 and PC3) 25, 26 as models to study arginine-starvation therapy. These cell lines are particularly sensitive to ADI treatment both *in vitro* and *in vivo*. In the short term, ADI- treated cells display general silencing of metabolic genes, including OXPHOS glycolysis and nucleotide synthesis genes, mitochondrial metabolite depletion, and mitochondrial dysfunction 23, 24, 26, 27. At the same time, mTOR activity is suppressed and protective autophagy is induced 25. Prolonged treatment (> 72 h) results in ROS production, excessive autophagy, and nuclear DNA leakage, leading to both chromatin-autophagy and mitophagy with eventual cell death 26, 27. In these models, ASS1 expression remains low throughout this period and cells are sensitive to arginine starvation.

In the present study, we set forth to identify pathways which lead to ADI resistance using breast MDA-MB-231 as a model. We have selected multiple resistant clones, referred to as ADIRs, and characterized their altered pathways as compared to the parental sensitive clones. We found that with no exception, ASS1 expression is elevated in all these clones, suggesting restoration of ASS1 is a necessary condition for resistance. We further established ASS1-overexpressing clones to study whether ASS1 is sufficient for ADI resistance. Interestingly, despite the high level of ASS1 expression in ASS1 over-expressor, they are consistently more sensitive to ADI than ADIRs both *in vitro* and* in vivo*, suggesting additional genetic or epigenetic changes have taken place in ADIRs. To look for these possible alterations which increase the resistance, we characterized the differences of the molecular signatures of ADIR and ASS1 over-expressor. We also took an unbiased approach, using CRISPR sg library to screen for genes whose depletions sensitized ADIR toward ADI. The two results converge, which identify the TREM1-CCL2 pathway as the critical component involved in resistance beyond the overexpression of ASS1. We have generalized and validated the results in prostate cancer cells, with similar results also obtained in a prostate cancer PC3 cell model. This work both uncovers a new pathway for ADI-resistance and provides additional targets to overcome ADI-resistance.

## Material and Methods

### Plasmids, shRNA, and reagents

Plasmid pCMV6-ASS1-Myc-DDK (CAT#: RC223189) was purchased from OriGene (USA). Plasmids pLX304-V5-TREM1 and pLX304-V5-CCL2 were purchased from Dharmacon Thermo Scientific (USA). Short hairpin RNAs (shRNAs) against human ASS1, TREM1, CCL2, AKT, and STAT3 were obtained from the National RNAi Core Facility (Academia Sinica, Taiwan). A control shRNA targeting Luciferase (Luc) was used as a negative control. The target sequences for specific genes are listed in Table S1. ADI-PEG20 was obtained from Polaris Pharmaceuticals (USA). AKT inhibitor, AKTi IV (CAS 681281-88-9), was purchased from Cayman Chemical (USA). CCR2 Antagonist (CAS 445479-97-0) was obtained from Santa Cruz Biotechnology (USA). CCL2 inhibitor, Bindarit (Synonyms: AF2838), was obtained from MedChemExpress (USA).

### Cell lines and establishment of ADI-resistance and stable clones

MDA-MB-231 and PC3 cells were obtained from Bioresource Collection and Research Center (Taiwan). CWR22Rv1, DU145, and BT549 cells were obtained from American Type Culture Collection Center (ATCC). MDA-MB-231 was cultured in DMEM (high glucose) medium, BT549 was cultured in DMEM/F12 medium, and PC3, CWR22Rv1, and DU145 were cultured in RPMI-1640 medium with 10% fetal bovine serum at 37 °C under 5% CO2. ADI-resistant clones were selected starting from 0.06 μg/mL ADI with 0.02 μg/mL increments per week in MDA-MB-231, PC3, CWR22Rv1, DU145, and BT549 cells. Resistant clones were routinely maintained at 0.9 μg/mL ADI-PEG20 for more than 3 months. ASS1-overexpressing cells in MDA-MBA-231 and PC3 cells were obtained by transfection of pCMV6-ASS1-Myc-DDK and following G418 selection (400 μg/mL; InvivoGen, USA). TREM1- and CCL2-overexpressing cells were generated by infection of lentivirus particles encoding TREM1-V5 and CCL2-V5, respectively, and following blasticidin selection (10 μg/mL; InvivoGen, USA). Single clones were selected by serial dilution in 96-well plates.

### Reverse transcription-quantitative PCR

Total RNA was extracted using TOOLSmart RNA Extractor reagent (BIOTOOLS, Taiwan), and cDNA synthesis was performed using ToolsQuant II Fast RT Kit (BIOTOOLS, Taiwan). Reverse transcription-quantitative PCR was performed in triplicate with TOOLS 2× SYBR qPCR Mix (BIOTOOLS, Taiwan) on the Rotor-Gene Q (Qiagen, USA). Relative expression levels were normalized to *RPLP0* gene. The sequences of primer sets designed to detect specific genes are listed in Table S2.

### Western blot analysis

Cells were lysed in RIPA buffer (Thermo Fisher Scientific, USA) containing a protease inhibitor cocktail (Roche, USA) and PMSF (Sigma, USA). Protein concentration was measured with a Bio-Rad Protein Assay Kit (Bio-Rad, USA). Primary antibodies used are listed in Table S3.

### Real-time Cell Analysis (RTCA) assay using the xCELLigence system

Tumor cells (6×10^3^) were seeded into 16-well E-plates (Acea Bioscience, USA) in 200 uL medium. After seeding, cells were monitored every 15 min by the xCELLigence® RTCA DP (Acea Bioscience, USA). After a 24 h cell attachment period, cells were treated with 0.15 μg/mL ADI. Real-time cell index and relative slopes (1/h) deduced from the proliferation curves were generated by RTCA software 2.0 (Acea Bioscience, USA).

### Cell viability assay

Tumor cells (3×10^3^) were seeded in a 96-well plate. The numbers of viable cells were counted using alamarBlue™ Cell Viability Reagent (Invitrogen; DAL1025). Absorbance was quantified by measuring the absorbance at 570 nm subtracted from the absorbance at 600 nm.

### Clonogenic assay

For the clonogenic assay, 1,000 cells/well were seeded in a 24-well plate in an appropriate culture medium. After a 24 h cell attachment period, cells were treated with 0.3 μg/mL ADI. Colonies were fixed with 100% methanol, stained with 0.25% crystal violet, and counted 2 weeks after plating.

### Flow cytometry analysis of apoptosis and cell cycle distribution

Apoptosis was detected by staining cells with Annexin V-FITC/propidium iodide (Merck-Millipore, Germany) according to the manufacturer's instructions. The cell cycle distribution analysis was measured by PI/RNase Staining Buffer (BD Pharmingen, USA). Data were acquired with the Attune NxT Flow Cytometer (Thermo Fisher, USA) and analyzed with FlowJo software (version 8.7.1).

### Enzyme-linked immunosorbent assay (ELISA)

CCL2 in cell culture supernatants were analyzed by ELISA kit (Biolegend, USA) according to the manufacturer's instructions.

### CRISPR/Cas9-mediated knockout

To knockout CCL2 in M231-ADIR and PC3-ADIR cells, sequences of single guide RNA (sgRNA) were designed from Feng Zhang's Lab (MIT) and cloned into the pAll-Cas9.Ppuro (National RNAi core, Academia Sinica, Taiwan). The following targeting sites were used: 5'- AGCGAGCCCTTGGGGAATGAAGG-3'. The sgRNA plasmids were separately transfected into M231-ADIR and PC3-ADIR cells using Lipofectamine 3000 reagent (Invitrogen; USA). At two days post-transfection, transfected cells were selected with 2 μg/mL puromycin for one week. Viable cells were limiting diluted into a 96-well plate for isolation of single cell clones. Knockout cells were confirmed by western blot analysis and DNA sequencing of the genomic regions.

### mRNA sequencing and TCGA datasets analysis

Total RNA was extracted from M231, M231-ADIR, and M231-ASS1 cells and sequenced using the Nextseq system according to the manufacturer's instructions. For mRNA sequencing, RNA quality and quantity were assessed by Bioanalyzer (Agilent, Bioanalyzer 2100 system, USA) and Qubit (Life Technologies, Qubit® 2.0 Fluorometer, USA), and then ligated to adaptor for further amplification (Illumina® TruSeq stranded mRNA, USA). All library preparation was performed at Taipei Medical University's Translational core facility. After sequencing was completed, reads file (fastq) were mapped to GRCh38 reference by STAR 28 and calculated for gene expression by RSEM 29. Differential genes were identified by R package, DESeq2 30, gene set enrichment analysis (GSEA), and Ingenuity Pathway Analysis (IPA) was used to decipherer gene function and network. The TCGA breast carcinoma cohort (TCGA-BRCA) gene expression dataset was downloaded using R package, TCGAbiolinks 31. All raw counts were normalized using DESeq2. To determine whether specific genes showed statistically significant differential expression between normal and tumor conditions and between histological types, a student t-test was used.

### Genome-wide CRISPR knockout screening and analysis

The human GeCKOv2 CRISPR knockout pooled library was a gift from Feng Zhang (Addgene #1000000049) and used to study genes responsible for ADI-resistance. Library A included 65,383 sgRNAs constructs and 3 sgRNAs per gene. Lentivirus for the GeCKOv2 Library A was prepared and titered to achieve MOI of 0.1-0.3 when transducing to M231-ADIR cells. Transduced cells were selected with puromycin at 2 μg/ml for 7 days to generate a baseline cell pool which was then treated with vehicle (PBS) and ADI (1 μg/mL) for 3 days. After treatment, PI+ cells were sorted by flow cytometry.

Genomic DNA was extracted from sorted cells using a Wizard® Genomic DNA Purification Kit (Promega, USA).

The sgRNA library was established using a two-step PCR process. The first step amplified the genomic DNA containing a sgRNA cassette sequence by specific primers. In the second step, Illumina sequencing adapters and barcodes were attached. All PCR reactions were performed using Herculase II Fusion DNA Polymerase (Agilent, USA). PCR products were purified and quantified with Qubit and Bioanalyzer, and libraries were sequenced on Illumina NextSeq500. After sequencing was finished, reads files (fastq) were trimming by specific sgRNA cassette sequence, then mapped to library A pool library sequence with Subread aligner 32. DESeq2 was used to calculate a normalized count value. R package, ggplot2, was used to draw scatter plot.

### Immunohistochemistry

Breast cancer tissue samples (n = 30) were obtained from the Shuang Ho Hospital (SHH) breast cancer cohort. Ethical approval for the study was obtained from Taipei Medical University's Joint Institutional Review Board (JIRB, approval number: N201603028). Tissue sections (4 µm) were deparaffinized and rehydrated in a gradually decreasing concentration of methanol (100%, 95%, and 70%). Antigen retrieval was carried out by boiling slides in a pressure cooker containing TrilogyTM buffer (Sigma-920P-06, Cell Marque, Sigma-Aldrich, Inc. USA) for 5 min, followed by incubation in a hydrogen peroxide blocking solution (TA-125-H2O2Q, Thermo Fisher Scientific, USA) for 10 min. Nonspecific binding was blocked with Ultra V Block (TA-125-PBQ, Thermo Fisher Scientific, USA) for 10 min. Slides were incubated in primary antibodies against TREM1 (ab225861, 1:200, Abcam Biotechnology, USA), CCL2 (ab73680, 1:200, Abcam Biotechnology, USA), CCR2 (ab176390, 1:200, Abcam Biotechnology, USA), and ASS1 (16210-1-AP, 1:200, Proteintech Group, USA) overnight at 4 °C. Then tissue slides were incubated in Primary Antibody Amplifier Quanto (TL-125-QPB, Thermo Fisher Scientific, USA) for 10 min, in Horseradish peroxidase (HRP) Polymer Quanto (TL-125-QPH, Thermo Fisher Scientific, USA) for 10 min and then in DAB Quanto Chromogen (TA-004-QHCX, Thermo Fisher Scientific, USA) diluted 3:100 in DAB Quanto Substrate (TA-125-QHSX, Thermo Fisher Scientific, USA) for 3 min. Slides were counterstained with hematoxylin. The immunoreactive score system (IRS) was used to measure protein of interest expression level as previously described 33.

### *In vivo* experiments

Tumor cells (2 × 10^6^) in serum-free medium were injected subcutaneously into the flank region of 6-week-old BALB/c nude mice. Intraperitoneal injection with vehicle versus ADI 11.5 mg/kg twice a week commenced two weeks following implantation. A caliper was used to measure tumor sizes weekly based on the formula: volume = 0.5236 × length × width × height. Mice were sacrificed 30 days after treatment. All experiments were approved by the Taipei Medical University Institutional Animal Care and Use Committee (approval number: LAC-2017-0247) and carried out under institutional guidelines and animal welfare standards.

### Statistical analysis

Statistical analysis involved the two-tailed Student *t* test or ANOVA using Prism software (GraphPad). Statistical significance is shown as *, *P* < 0.05; **, *P* < 0.01; and ***, *P* < 0.001.

## Results

### Development and characterization of ADI-resistant clones

**Figure 1:**
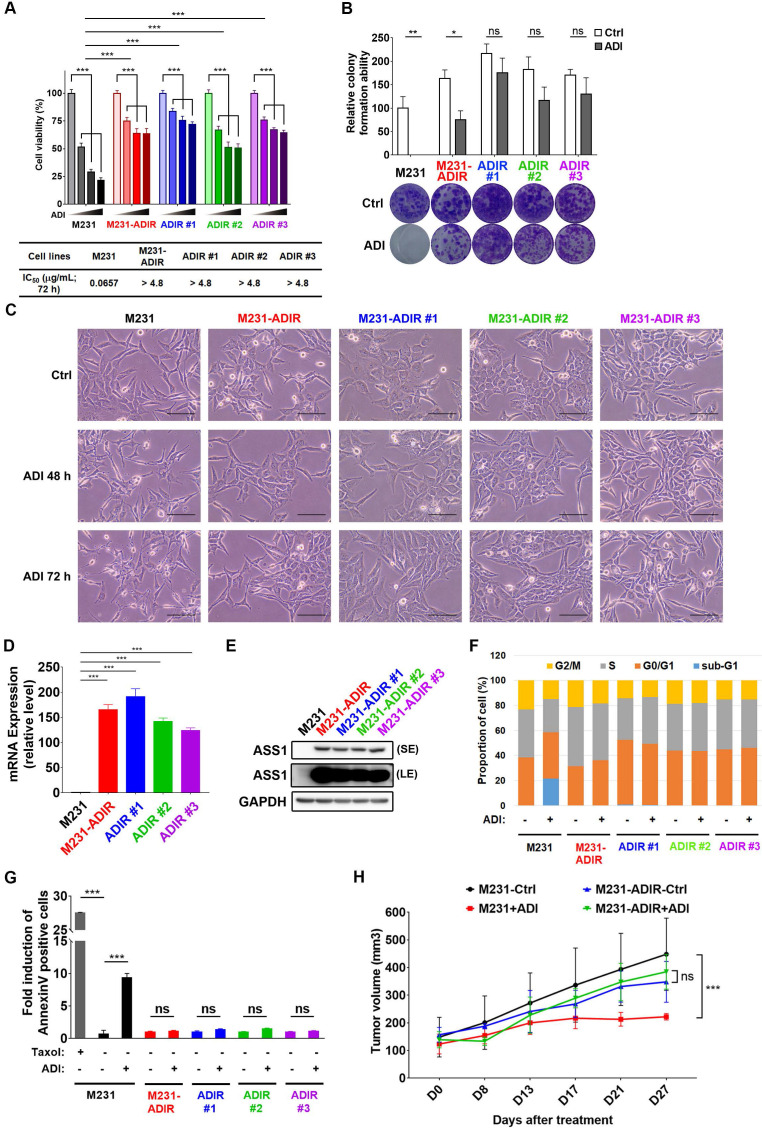
** Development of ADI-PEG20 resistant cells.** A, Parental MDA-MB-231 and its ADI-resistant variants (M231-ADIR, M231-ADIR #1, M231-ADIR #2, and M231-ADIR #3) were treated with an increasing dose of ADI (0, 0.075, 0.3, or 1.2 µg/mL) for 3 days. Cell viability of treated cells was measured by MTS assay. IC_50_ was calculated and listed. B, Clonogenic assay performed in indicated cells treated with vehicle or ADI at 0.3 µg/mL for 14 days. Top panel: Colonies were quantified as percentage inhibition of colony formation. Bottom panel: Representative clonogenic plates were photographed. C, Conventional light microscopy of indicated cells exposed to vehicle or 0.3 µg/mL ADI for 48 h or 72 h. Scale bar, 100 µm. D, mRNA levels of *ASS1* in indicated cells were measured by real-time PCR. E, Immunoblotting assays for ASS1 (LE, long exposure; SE, short exposure) and GAPDH in indicated cells. F, Flow cytometry analysis of the effect of ADI treatment (0.3 μg/mL) on the cell cycle in indicated cells for 3 days. Data shown represent quantification of cell cycle analysis. G, Apoptosis was analyzed by flow cytometry for Annexin V. Data shown represent mean ± SD (percentage of apoptotic cells relative to vehicle control) in indicated cells. H, M231 and M231-ADIR cells were injected subcutaneously into BALB/c nude mice. Intraperitoneal injection with vehicle versus ADI 11.5 mg/kg twice a week was commenced two weeks following implantation. Tumor volume of mice xenograft was monitored at indicated time points. All data are shown as mean ± SD of triplicate measurements, with *P* values from the Student *t*-test. ***, *P* < 0.001.

To investigate the mechanism of resistance, we cultured triple negative breast cancer MDA-MB-231 cells with incrementally increasing concentrations of ADI-PEG20 (abbreviated as ADI in this article) starting from 0.06 μg/mL. Once cells developed resistance to ADI, multiple independently derived clonal cells were isolated, (e.g., M231-ADIR #1, M231-ADIR #2, and M231-ADIR #3) from the mixed population (M231-ADIR). MTT and clonogenic assays indicated either the mixed population or the three independent clones were more resistant to ADI than their parental cell lines (M231) in both cell viability (Figure 1A) and colony formation (Figure 1B) assays. The IC_50_ of ADI for the parental sensitive cells is 0.07 μg/mL, whereas those of the resistant cells are larger than 4.8 μg/mL. Upon ADI treatment at 0.3 μg/mL, the MDA-MB-231 parental cell line underwent morphological changes visible after 48 h of treatment (Figure 1C) with cells showing the roundness and smaller in size characteristic of dying cells. By contrast, the ADIR cells, either the pool or individual clones, did not have obvious morphological changes.

We then analyzed the expression of ASS1 in these cell lines and found that indeed all these cell lines have significantly higher ASS1 expression levels than parental M231 at both transcript and protein levels (Figure 1D-E), indicating that restoration of ASS1 expression is a key component of ADI-resistance. Consistent with this notion, knockdown of ASS1 from M231-ADIR resensitized the cell lines toward ADI (Figure S1A-C).

ADI treatment of ASS1-low tumor cells leads to cell cycle arrest and apoptosis (shown as sub-G1 fraction) in various tumor models 15, 25, 34. As shown in Figure 1F and S1D, ADI treatment of M231 parental cell lines gave rise to significant sub G1 fraction (blue) and shortened S (gray) and G2M (yellow) phases. However, the M231-ADIR pool and three independent ADIR clones display a cell cycle pattern similar to untreated parental cells upon ADI treatment. Using flow cytometry, we also directly measured apoptosis/necrosis induction (Annexin V/Propidium iodide positive) by ADI treatment on the parental and resistant cells. As shown in Figure 1G and S1E, parental cells are highly sensitive to ADI treatment and exhibit cell death characteristics. In contrast, the M231-ADIR and individual clones are all refractory to cell death.

To validate the ADI resistance of ADIRs *in vivo*, M231 parental and M231-ADIR cells were injected subcutaneously into nude mice and tumor volumes were monitored over time. Under ADI treatment, the growth of M231 parental cells was significantly inhibited, whereas growth of M231-ADIR did not change significantly (Figure 1H).

### Development of ASS1-overexpressing clones

**Figure 2:**
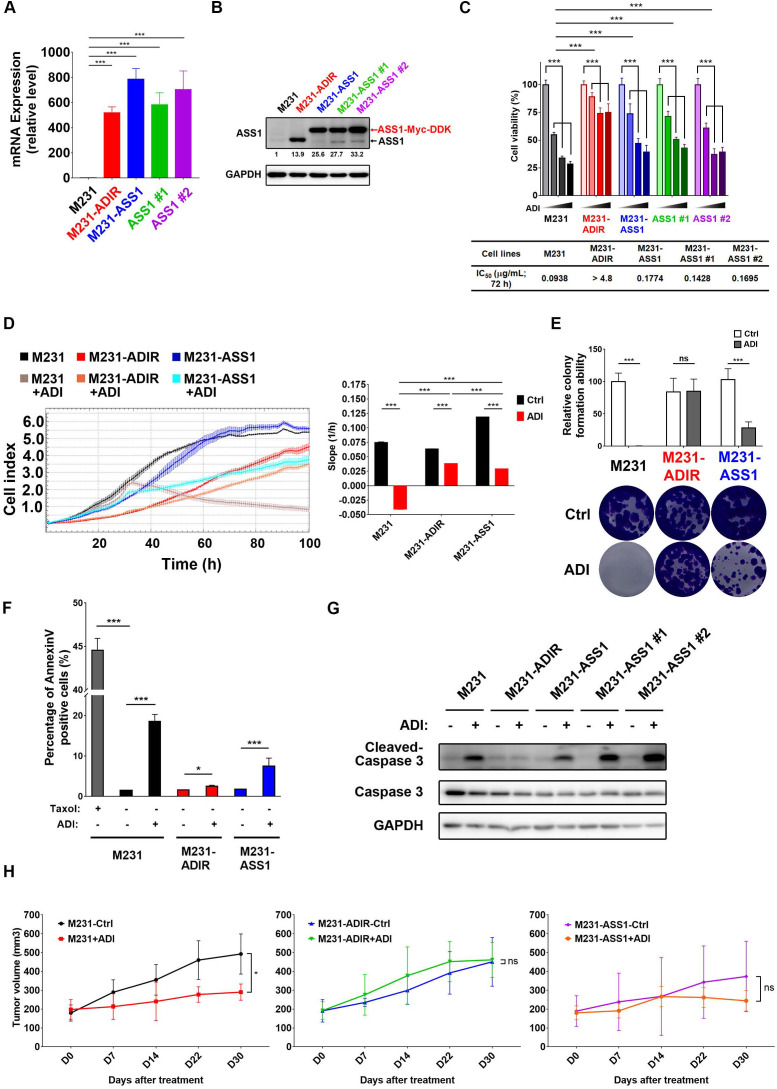
** ASS1 overexpression is not sufficient for ADI-resistance.** A, mRNA levels of *ASS1* in parental M231, M231-ADIR, and ASS1-overexpressing (M231-ASS1, M231-ASS1 #1, and M231-ASS1 #2) cells were measured by real-time PCR. B, Immunoblotting assays for ASS1 and GAPDH in indicated cells. The protein bands were quantitated by densitometry, expressed relative to GAPDH, and normalized to the parental M231 cells. C, Indicated cells were treated with an increasing dose of ADI (0, 0.075, 0.3, or 1.2 µg/mL) for 3 days. Cell viability of treated cells was measured by MTS assay. IC_50_ was calculated and listed. D, Cell proliferation of indicated cells treated with vehicle or ADI at 0.15 µg/mL at 24 h were measured by RTCA assay. Left panel: real-time cell index generated by RTCA software. Right panel: slope of the line between the 40 and 60 h interval (changes in cell index/hour). E, Clonogenic assay performed in indicated cells treated with vehicle or ADI at 0.3 µg/mL for 14 days. Top: Representative clonogenic plates were photographed. Bottom: Colonies were quantified as percentage inhibition of colony formation. F, Apoptosis was analyzed by flow cytometry for Annexin V. Data shown represent mean ± SD (percentage of apoptotic cells relative to vehicle control) in indicated cells. G, Immunoblotting assays of cleaved caspase-3, pro-caspase-3, and GAPDH in indicated cells upon treatment of ADI at 0.3 µg/mL for 48 h. H, M231, M231-ADIR, and M231-ASS1 cells were injected subcutaneously into BALB/c nude mice. Intraperitoneal injection with vehicle versus ADI 11.5 mg/kg twice a week was commenced two weeks following implantation. Tumor volume of mice xenograft was monitored at indicated time points. All data are shown as mean ± SD of triplicate measurements, with *P* values from the Student *t*-test. *, *P* < 0.05; **, *P* < 0.01; ***, *P* < 0.001; n.s., not significant.

Our data presented above have provided strong evidence that ASS1 expression is necessary to confer ADI-resistance. It is not clear, however, whether or not ASS1 is sufficient for resistance. To this end, we established stable ASS1-expressing clones of MDA-MB-231 cells (M231-ASS1) and two independent clonal cells, namely M231-ASS1 #1, and M231-ASS1 #2, from the mixed population. This pooled population expresses ASS1 transcripts and proteins at higher levels than M231-ADIR (Figure 2A-B). The higher molecular weight of ASS1 in M231-ASS1 is due to the Myc-DDK epitope tag. Based on MTT (Figure 2C), Real-Time Cell Analysis (RTCA) (Figure 2D) and clonogenic assays (Figure 2E), M231-ASS1 (ADI IC_50_ = 0.18 μg/mL) was indeed more resistant to ADI treatment than the parental cell line (ADI IC_50_ = 0.09 μg/mL). Interestingly, despite a comparable level of ASS1, M231-ASS1 is considerably more sensitive to ADI than M231-ADIR (ADI IC_50_ > 4.8 μg/mL). In addition, like the parental cell line, M231-ASS1 is sensitive to ADI induced apoptosis, whereas M231-ADIR is more resistant, based on both flow cytometry (Figure 2F & S1F) and western blot analyses of caspase-3 cleavage (Figure 2G). In the xenograft model, M231-ASS1 did not achieve the same level of ADI resistance as does M231-ADIR and remains somewhat sensitive to ADI (Figure 2H). Immunohistochemical staining (IHC) analysis of excised tumors further confirmed that the expression levels of ASS1 were higher in M231-ASS1 and M231-ADIR tumors as compared to the parental one (Figure S2A-B). Collectively, these results suggest that factors in addition to ASS1 contribute to the ADI-resistance of M231-ADIR.

### Molecular pathways associated with ADI-resistance

**Figure 3:**
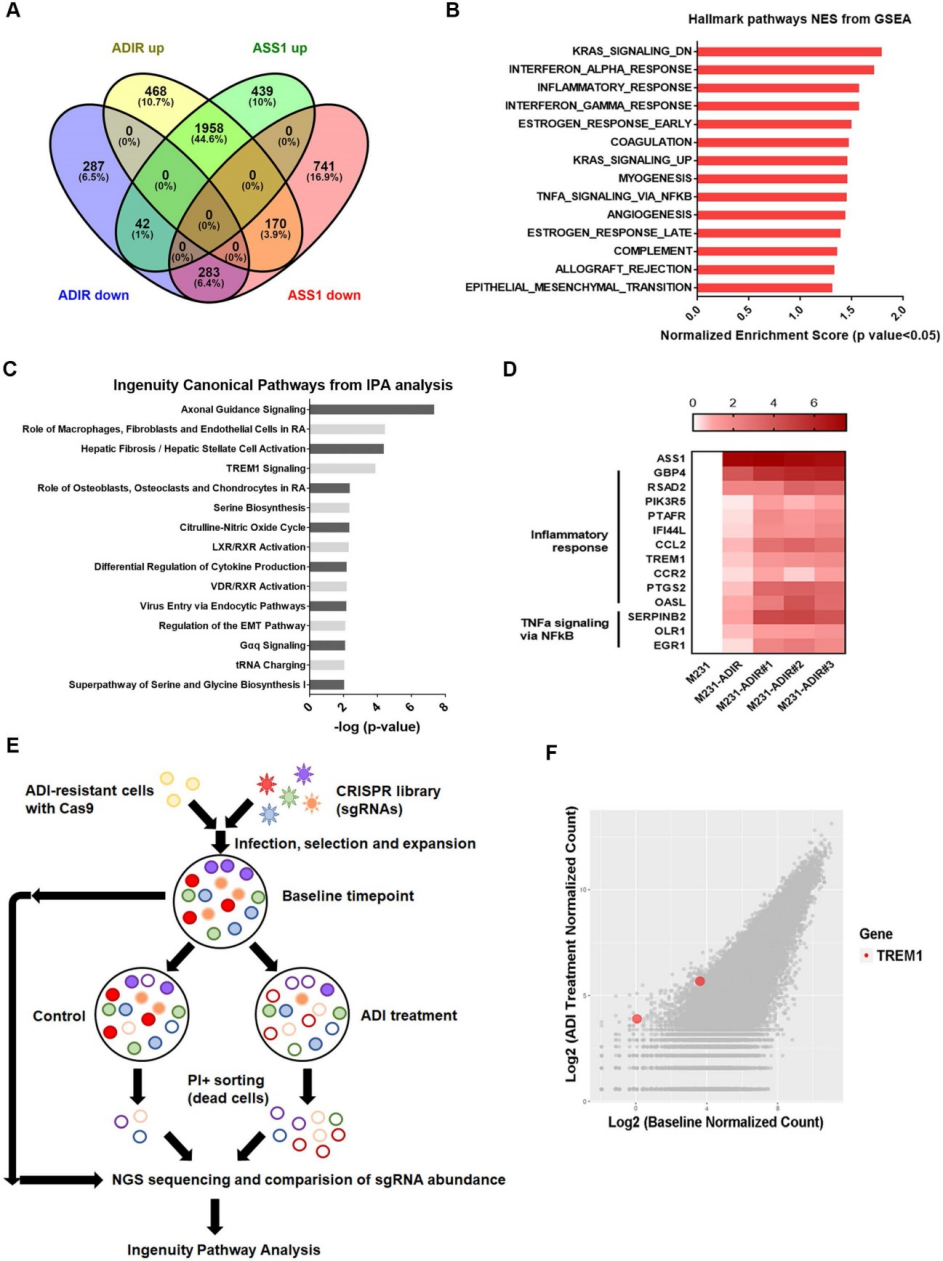
** Immune response is upregulated in ADI-resistance.** A, Venn diagram indicating overlapping differentially expressed gene (DEG) sets among ADIR-up (M231-ADIR vs. M231, upregulated), ADIR-down (M231-ADIR vs. M231, downregulated), ASS1-up (M231-ASS1 vs. M231, upregulated), and ASS1-down (M231-ASS1 vs. M231, downregulated). B, DEGs significantly upregulated in M231-ADIR versus M231 cells analyzed using Hallmark pathway datasets in GSEA. NES (normalized enrichment score) and p-values are given as a bar graph. C, Ingenuity Pathway Analysis. Top ranked canonical pathways (-log(p-value) > 1.30) are shown. D, Heat map representing genes based on expression levels in indicated cells by real-time PCR assay. Levels of up-expression (red) are shown. E, Schematic representation of genome-wide human GeCKO knockout screening in M231-ADIR cells with and without ADI treatment. After next-generation sequencing of the pooled library, the frequency of each sgRNA in baseline and PI+ sorted cells were counted. F, Scatterplot of fold change of sgRNA scores of sorted dead cells after ADI treatment versus baseline cells. sgRNAs targeting TREM1 were marked as red.

To understand the molecular basis underlying the difference between ASS1-overexpressing and ADI-resistant cells, we carried out gene expression profiling through RNA sequencing. As shown in Figure 3A, analysis revealed that 2596 genes were up-regulated and 612 genes were down-regulated at least two-fold in M231-ADIR cells relative to their parental M231 cells, while 2439 genes were up-regulated and 1194 genes were down-regulated in M231-ASS1 cells relative to M231 cells. There were 1958 up-regulated and 283 down-regulated genes that overlapped in M231-ADIR and M231-ASS1 cells relative to M231 cells.

To identify the mechanisms associated with the higher level of resistance found in M231-ADIR but not in M231-ASS1, Gene Set Enrichment Analysis (GSEA) was used to analyze up-regulated genes in M231-ADIR cells. This analysis revealed upregulation of inflammatory response and signaling pathways including K-RAS, TNFα, and epithelial-mesenchymal transition, which are distinct from those of M231-ASS1 (Figure 3B & S3A). Ingenuity Pathway Analysis (IPA) was also used to analyze upregulated genes in M231-ADIR cells (Figure 3C), many of which are involved in immune response pathways, including leukocyte extravasation signaling, IL-8 signaling, and TREM1 signaling.

Real-time q-PCR was used to validate the M231-ADIR RNA-seq data and extend the analysis to individual ADIR clones (Figure 3D). In agreement, the expression of inflammatory response and TNFα signaling genes was found to be elevated. Among these inflammatory response genes, TREM1 and CCL2 will be described in further detail below.

### Genome-wide CRISPR knockout to identify genes contributing to resistance

To identify genes critical for ADI resistance, we performed a genome-wide CRISPR/Cas9 knock-out screening by using human GeCKO v2.0 single-guide RNA (sgRNA) pooled library (Figure 3E). After stable incorporation of the Cas9 gene, M231-ADIR cells were transduced with lentiviruses expressing the pooled GeCKO Library A at an MOI of 0.3, followed by puromycin selection. We then treated the resistant cell pool with vehicle or ADI for 3 days to sort the dying portions by flow cytometry. PI (propidium iodide)+ sorted cells were harvested and their corresponding sgRNAs were determined by next-generation sequencing. The results revealed a subset of sgRNAs targeting 265 genes which were significantly increased in ADI-treated cells when compared to the untreated control. These genes whose absence caused death of ADIR in the presence of ADI are likely candidates for ADI resistance factors.

**Figure 4:**
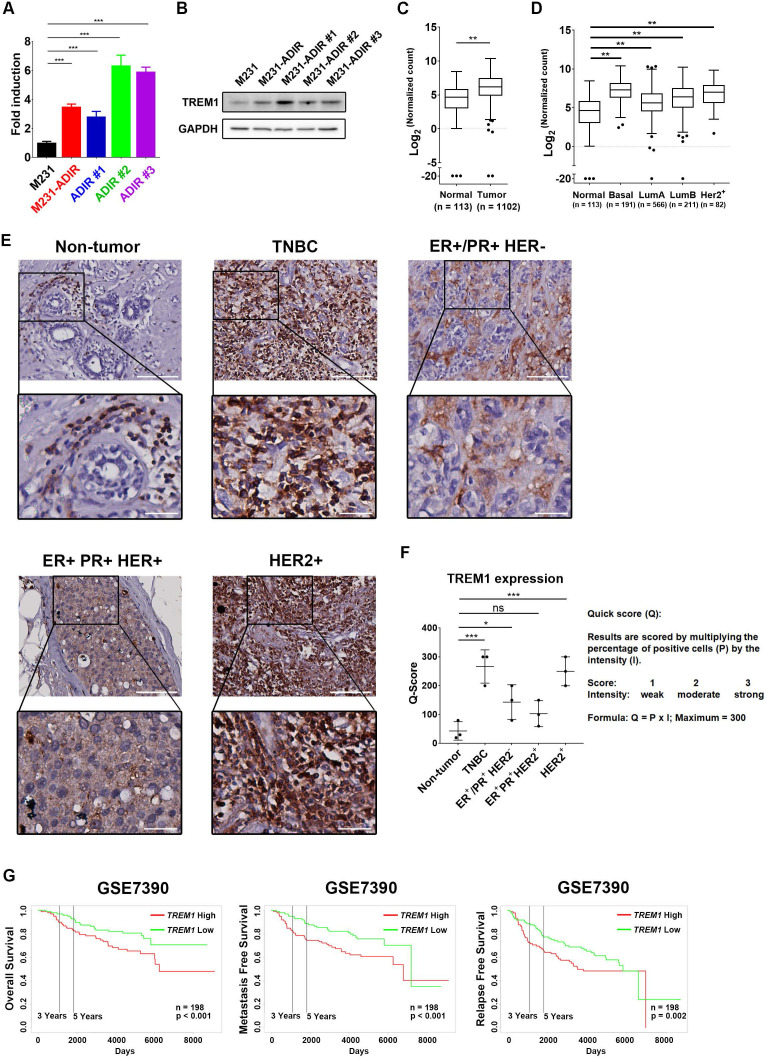
** Participation of TREM1 in breast cancers.** A, mRNA levels of TREM1 were measured in indicated cells by real-time PCR. B, Immunoblotting assays for TREM1 and GAPDH in indicated cells. C, Expression levels of TREM1 in breast carcinoma (n = 1102) and normal (n = 113) tissue in TCGA-BRCA cohort. D, Expression levels of TREM1 in different breast cancer subtypes in TCGA-BRCA cohort. Number of samples in each subtype is indicated at the bottom. E, Representative IHC staining of TREM1 signal in different breast cancer subtypes and normal tissue. Scale bar, 100 µm in the original region and 25 µm in the zoomed-in region respectively. F, Intensity of TREM1 signals was quantified as Quick score (Q). G, Kaplan-Meier analysis of overall survival (OS), metastasis-free survival (MFS), and relapse-free survival (RFS) curves of patients with breast cancer (GSE7390; n = 198) stratified by TREM1 expression.

Among the list of genes, triggering receptor expressed on myeloid cells 1 (TREM1), whose sgRNA was one of the most enriched ones in PI+ cells upon ADI treatment, was identified (red dots, Figure 3F). TREM1, a potent amplifier of inflammatory responses, is upregulated in M231-ADIR and individual ADIR clones according to real-time PCR (Figure 4A), western blot analysis (Figure 4B), and IHC of xenograft tumors (Figure S2A & S2C).

TREM1 was originally discovered as a surface receptor on myeloid cells and triggers signal responses. Interestingly, TREM1 is aberrantly overexpressed in breast cancer tissue as compared to adjacent normal tissue, based on the expression profiles of breast cancer cohort from The Cancer Genome Atlas (TCGA) (Figure 4C). TREM1 is differentially up-regulated in all breast cancer subtypes, especially in Basal-like (BL) breast cancers, and HER2-positive (HER+) breast cancers (Figure 4D). To further confirm the TREM1 expression pattern in breast cancers, we performed IHC analysis for TREM1 in breast cancer and non-tumor tissue samples. Indeed, TREM1 expression was stronger in breast cancer tissue compared to normal tissue (Figure 4E & 4F). Moreover, Kaplan-Meier analysis using publicly available expression datasets revealed that high TREM1 expression was associated with a poor clinical outcome, including overall survival (OS), metastasis-free survival (MFS), and relapse-free survival (RFS) (Figure 4G & S4A).

### TREM1 contributes to ADI resistance

**Figure 5:**
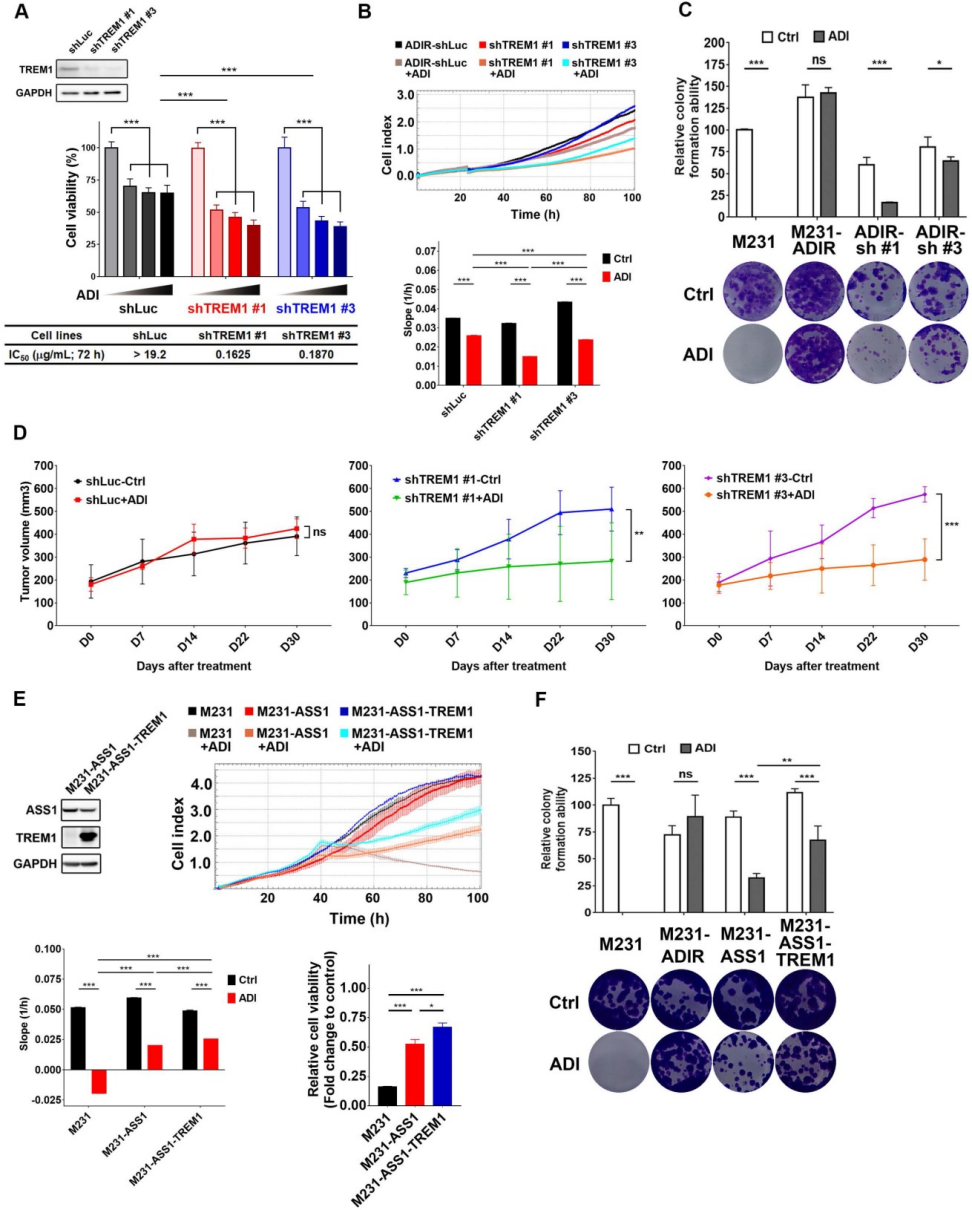
** Participation of TREM1 in ADI-resistance.** A, M231-ADIR cells infected with lentiviral vectors encoding shLuc and shTREM1. #1 and #3 indicate distinct shRNAs targeting different regions within TREM1. Knock-down efficiency was checked by immunoblotting assays. Indicated cells were treated with increasing dose of ADI (0, 0.15, 0.3, or 0.6 µg/mL) for 3 days. Cell viability of treated cells was measured by MTS assay. IC_50_ was calculated and listed. B, Cell proliferation of indicated cells treated with vehicle or ADI at 0.15 µg/mL at 24 h were measured by RTCA assay. Top panel: real-time cell index generated by RTCA software. Bottom panel: slope of the line between the 40 and 60 h interval (changes in cell index/hour). C, Clonogenic assay performed in indicated cells treated with vehicle or ADI at 0.3 µg/mL for 14 days. Top panel: Colonies were quantified as percentage inhibition of colony formation. Bottom panel: Representative clonogenic plates were photographed. D, Indicated cells were injected subcutaneously into BALB/c nude mice. Intraperitoneal injection with vehicle versus ADI 11.5 mg/kg twice a week was commenced two weeks following implantation. Tumor volume of mice xenograft was monitored at indicated time points. E, Top left: immunoblotting assays for ASS1, TREM1, and GAPDH in indicated cells. Top right: cell proliferation of indicated cells treated with vehicle or ADI at 0.15 µg/mL at 24 h was measured by RTCA assay. Real-time cell index generated by RTCA software. Bottom left: slope of the line between the 40 and 60 h interval (changes in cell index/hour). Bottom right: relative cell viability to vehicle control after 72 h treatment. F, Clonogenic assay performed in indicated cells treated with vehicle or ADI at 0.3 µg/mL for 14 days. Top panel: Colonies were quantified as percentage inhibition of colony formation. Bottom panel: Representative clonogenic plates were photographed. All data are shown as mean ± SD of triplicate measurements with P values from the Student t test. *, *P* < 0.05; **, *P* < 0.01; ***, *P* < 0.001; n.s., not significant.

To determine whether TREM1 is required for ADI-resistance, TREM1 was knocked down using two shRNAs against TREM1 in M231-ADIR cells (Figure 5A & S3B). Based on MTT (Figure 5A), RTCA (Figure 5B), and clonogenic assays (Figure 5C), cells carrying shRNA targeting TREM1 showed significantly lower cell viability than those with control shRNA in the presence of ADI.

We next examined whether TREM1 is crucial for resistance to ADI *in vivo*. TREM1-silenced cells were injected into nude mice, and the tumor volume in vehicle-control group and ADI treatment group was monitored (Figure 5D). IHC analysis of implanted tumors confirmed that the expression levels of TREM1 were lower in TREM1-silenced tumors (Figure S4B-C). These results demonstrate that knockdown of TREM1 markedly attenuated tumor growth upon ADI treatment, suggesting that TREM1 plays an important role in ADI-resistance.

We further asked whether overexpression of TREM1 in M231-ASS1, which does not express TREM1, would elevate its resistance to ADI. This was indeed the case, based on RTCA (Figure 5E) and clonogenic assay (Figure 5F). Overexpression of TREM1 alone in parental cells, however, does not confer resistance to ADI (Figure S4E-F), suggesting TREM1 is a necessary but not sufficient factor for ADI resistance. Collectively, these data suggest that TREM1 plays an auxiliary role to ASS1 in inducing full ADI-resistance.

### TREM1 signals are activated in ADI-resistant cells

**Figure 6:**
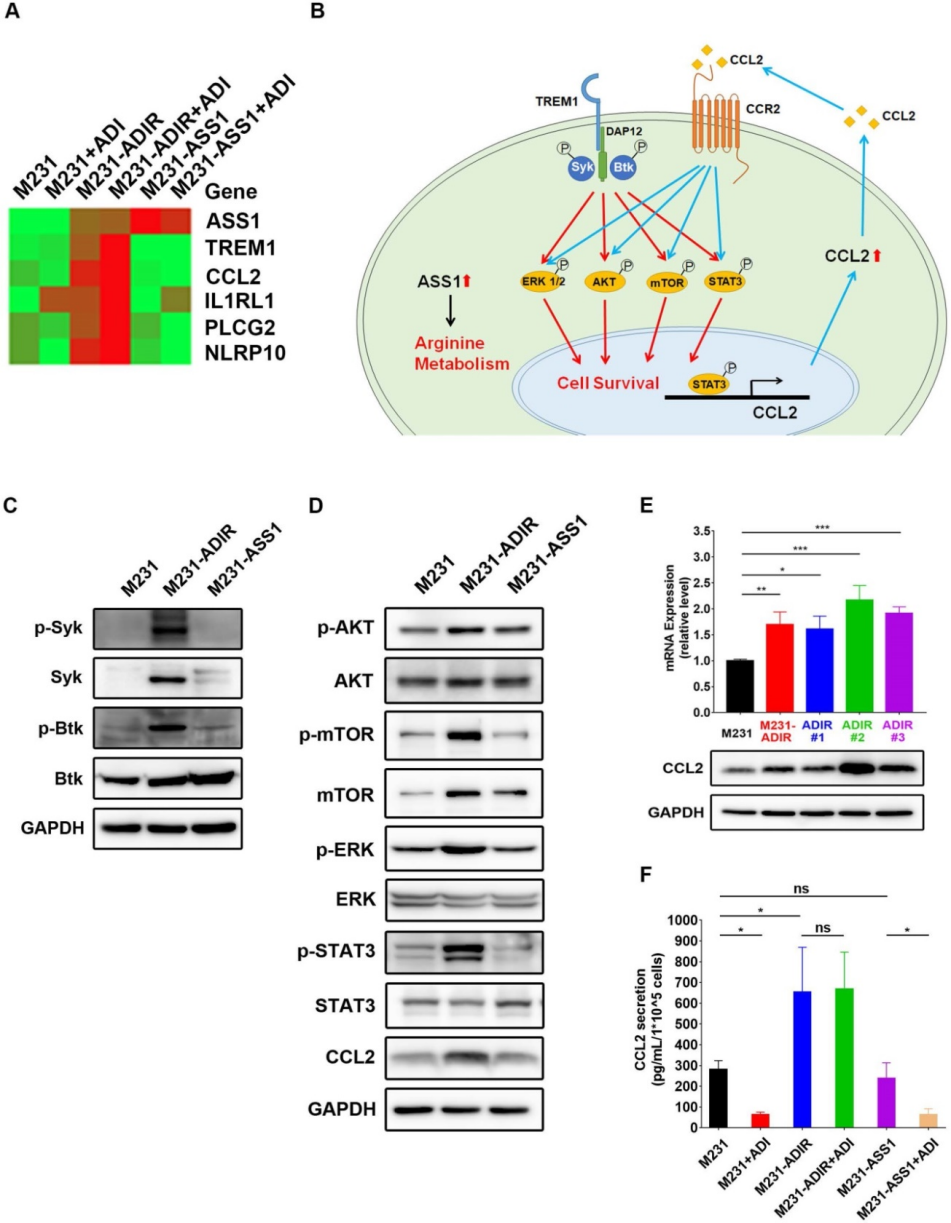
** TREM1 promotes ADI-resistance by activating AKT and mTOR signaling pathways.** A, Heat map representing genes based on expression levels in indicated cells with vehicle or ADI at 0.3 µg/mL for 48 h. Levels of down-expression (green) or up-expression (red) are shown. B, Schematic presentation of AKT/ERK/mTOR/STAT3 signaling pathways involved in ADI-resistance. Blue lines represent positive feed-back loops exerted by CCL2. C, Immunoblotting assays for phospho-Syk, Syk, p-Btk, Btk, and GAPDH in indicated cells. D, Immunoblotting assays of protein expressions in indicated cells. E, mRNA (top panel) and protein (bottom panel) levels of CCL2 were measured by real-time PCR and immunoblotting assays respectively in indicated cells. F, Secreted CCL2 in indicated cells were measured by ELISA assay. All data are shown as mean ± SD of triplicate measurements, with P values from the Student *t*-test. *, *P* < 0.05; **, *P* < 0.01; ***, *P* < 0.001; n.s., not significant.

If TREM1 signaling is important for ADI resistance, we expect genes involved in TREM1 pathway to be elevated in ADIR cells. Real-time PCR analysis revealed that expression of TREM1 pathway components (defined by IPA) including CCL2, IL1RL1, PLCG2, and NLRP10 are all upregulated in M231-ADIR pool and individual clones (Figure 6A). By contrast, M231-ASS1 cells did not display such upregulations.

TREM-1 is a membrane-associated receptor which transmits signals via adapter protein DAP12 and tyrosine kinases such as Syk and Btk 35, 36 (Figure 6B). This is followed by activation of ERK, PI3K/AKT/mTOR, and phosphorylation of STAT3, which leads to the transcriptional activation of pro-inflammatory chemokines (e.g., CCL2) 37. While most previous studies have been carried out in myeloid cells, we were interested in whether these signal pathways were also activated in ADIR.

Among the different tyrosine kinases tested, activation of Syk and Btk were observed as reflected by their tyrosyl phosphorylations (Figure 6C). The level of Syk, usually low in breast cancer cells, was upregulated. The ensuing signal cascades including the phosphorylation of AKT, mTOR, ERK, and STAT3 were also activated in M231-ADIR cells (Figure 6D). Consistently, both mRNA and protein levels of chemokine CCL2 were up-regulated in M231-ADIR as well as in the individual clones (Figure 6E). ELISA assay also confirmed the increased secretion of CCL2 in M231-ADIR cells, but not in M231-ASS1 cells. Furthermore, the expression levels of TREM1 and CCL2 correlate well in IHC staining of xenograft tumors (Figure S2A & S2D). Upon treatment of ADI, the secretion of CCL2 was down-modulated in parental M231 and M231-ASS1 cells, but not affected in M231-ADIR cells (Figure 6F). The abundant level of secreted CCL2 may play an autocrine or paracrine role in sustaining the growth of resistant cells (see below).

To generalize the significance of TREM1/CCL2 axis in ADI resistance, another ASS1-deficient BT-549 breast cancer cell line was used to establish ADI-resistant clones (Figure S5A). In this cell line too, the RNA expressions of TREM1 and CCL2 were up-regulated in the resistant clones (Figure S5B).

### CCL2 as a downstream effector of TREM1 contributes to ADI resistance

**Figure 7:**
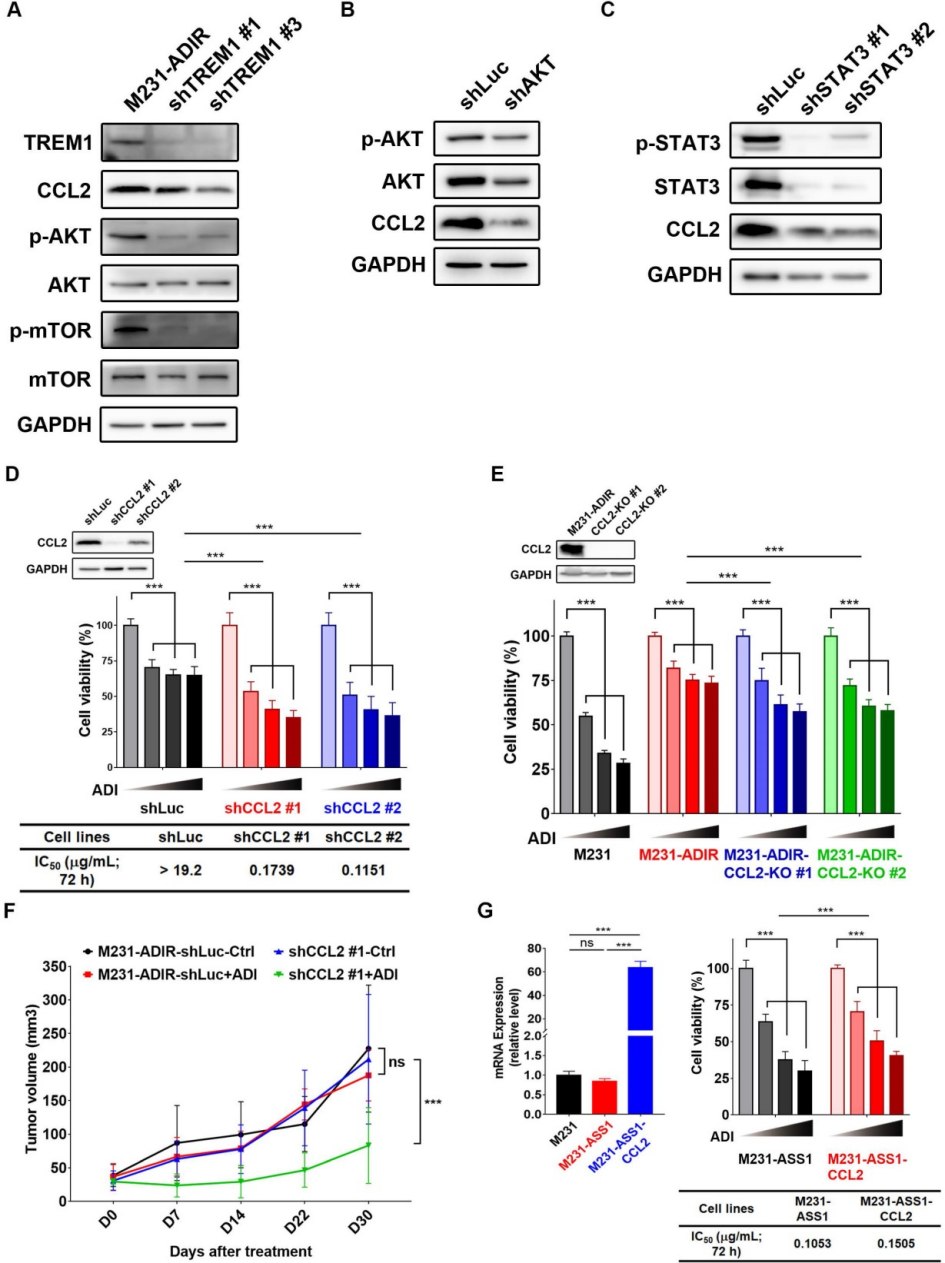
** CCL2 contributes to ADI-resistance as a downstream target of TREM1.** A, Immunoblotting assays for TREM1, CCL2, phospho-AKT, AKT, p-mTOR, mTOR, and GAPDH in TREM1-silenced M231-ADIR cells. B, Immunoblotting assays for phospho-AKT, AKT, CCL2, and GAPDH in AKT-silenced M231-ADIR cells. C, Immunoblotting assays for phospho-STAT3, STAT3, CCL2, and GAPDH in STAT3-silenced M231-ADIR cells. D, M231-ADIR cells infected with lentiviral vectors encoding shLuc and shCCL2. #1 and #2 indicate distinct shRNAs targeting different regions within CCL2. Knock down efficiency was checked by immunoblotting assays. Indicated cells were treated with increasing dose of ADI (0, 0.15, 0.3, or 0.6 µg/mL) for 3 days. Cell viability of treated cells was measured by MTS assay. E, Immunoblotting assays were used to validate the expression of CCL2 in CCL2-knockout forms (KO #1 and KO #2). Indicated cells were treated with increasing doses of ADI (0, 0.075, 0.3, or 1.2 µg/mL) for 3 days. Cell viability of treated cells was measured by MTS assay. F, Indicated cells were injected subcutaneously into BALB/c nude mice. Intraperitoneal injection with vehicle versus ADI 11.5 mg/kg twice a week was commenced two weeks following implantation. Tumor volume of mice xenograft was monitored at indicated time points. G, Left panel: mRNA level of CCL2 by real-time PCR assay. Right panel: Indicated cells were treated with increasing dose of ADI (0, 0.075, 0.15, or 0.3 µg/mL) for 3 days. Cell viability of treated cells was measured by MTS assay. IC_50_ was calculated and listed. All data are shown as mean ± SD of triplicate measurements, with P values from the Student t test. *, *P* < 0.05; **, *P* < 0.01; ***, *P* < 0.001; n.s., not significant.

As shown above, CCL2 is upregulated in ADI resistant cells. CCL2, monocyte chemoattractant protein 1 (MCP-1/CCL2), is recognized for its role involved in cancer progression and drug resistance 38-40. To demonstrate the functional relevance of TREM1 signaling and CCL2 upregulation, we depleted individual TREM1 signaling component in M231-ADIR cells. Knockdown of TREM1 suppressed CCL2 expression at transcript (Figure S6A), protein (Figure 7A), which is accompanied by the suppression of AKT and mTOR activation. Likewise, knockdown of AKT and STAT3 down-regulated the expression of CCL2 in M231-ADIR cells (Figure 7B & 7C). These studies confirm the signal cascade illustrated in Figure 6B. In addition, the expression patterns of CCL2 and its receptor CCR2 were similar to that of TREM1 through IHC staining in breast cancer and non-tumor tissue samples (Figure S7 & S8). These data together suggest that CCL2 is a critical downstream effector of TREM1.

To determine the role of CCL2 to ADI-resistance, we generated CCL2-silencing (Figure 7D & S6B) and CCL2-knockout (Figure 7E) M231-ADIR cells and confirmed CCL2 expression by real-time PCR and immunoblot analysis. Both CCL2 knock-down and knockout clones' viability was significantly decreased upon ADI treatment as compared to control cells. In xenograft studies, CCL2-silenced M231-ADIR cells significantly attenuated tumor growth upon ADI treatment (Figure 7F). IHC analysis of implanted tumors further confirmed that the expression level of CCL2 was lower in CCL2-silenced tumors (Figure S6C & S6E).

In addition, silencing of CCL2 diminished phosphorylation of AKT, suggesting that CCL2 contributes significantly to AKT activation (Figure S6F). Meanwhile, inhibition of AKT by its inhibitor, AKTi IV, overcame ADI resistance (Figure S6G). These data together suggest that CCL2 is a critical downstream effector of TREM1 and plays an important role in ADI resistance at least in part by AKT activation.

Consistently, co-expression of CCL2 and ASS1 significantly increased cell viability upon ADI treatment in MTT assay (Figure 7G). Based on these data, we hypothesize that ASS1 expression restores arginine metabolism in M231-ADIR cells, whereas TREM1 expression stimulates AKT/mTOR/STAT3 and a CCL2 feed-forward loop to provide survival signals contributing to ADI resistance in breast cancer cells (Figure 6B).

### TREM1/CCL2 also contributes to ADI resistance in prostate cancer

To generalize our observations to prostate cancer cells, we developed ADI-resistant clones (PC3-ADIR) and ASS1-overexpressing clones (PC3-ASS1) in prostate cancer PC3 cell line.

Similar results were obtained: namely, PC3-ADIR is more resistant than PC3-ASS1 (Figure S9A). In PC3-ADIR, CCL2 expression was highly upregulated both at the transcript and protein levels (Figure S9B-C). A significantly increased level of secreted CCL2 could be identified in PC3-ADIR as compared to PC3 (Figure S9D). The expression of TREM1 remains similar, in PC3-ADIR, although its overexpression in PC3 does increase CCL2 expression and the ADI resistance (Figure S9E-F). These data suggest CCL2 overexpression is the predominant factor in PC3-ADIR resistance. Moreover, knock-out of CCL2 sensitized PC3-ADIR toward ADI (Figure S9G-K) in PC3-ADIR cells according to MTT, RTCA, and clonogenic assays. Finally, increased phosphorylation of AKT and mTOR was also observed in PC3-ADIR cells, but not in PC3-ASS1 cells (Figure S9L).

We have characterized two additional ASS1-low castration-resistant prostate cancer cell lines, CWR22Rv1 and DU145 and isolated their ADI-resistant derivatives (Figure S5C & S5E). These resistant clones all had upregulated ASS1 expression. The RNA expression of TREM1 was up-regulated in CWR-ADIR cells, but not in DU145-ADIR, indicating there are additional TREM1/CCL2 independent pathways associated with full resistance to ADI (Figure S4D & S4F). Altogether, among the five cell lines we studied (two breast and three prostate cancers), four resistant clones have elevated levels of TREM1 or CCL2. These data suggest that TREM1/CCL2 axis activation conferring full ADI-resistance is a dominant, but not universal, mechanism.

### New therapeutic strategies: TREM1/CCL2/CCR2 as a therapeutic target

The upregulation of TREM1/CCL2 in ADI resistant clones and their knockdowns overcoming resistance provide opportunities for potential therapeutic intervention. To this end, we tested the CCR2 antagonist (CAS 445479-97-0) and clinically approved CCL2 inhibitor, Bindarit, for treatment of M231-ADIR and PC3-ADIR cells. As shown in Figure S4G-S4I, the growths of both ADI-resistant cell lines were inhibited, raising the possibility of application of inhibitors to the TREM1/CCL2/CCR2 axis to circumvent ADI resistance.

## Discussion

Arginine-deprivation therapy for cancer has received increasing attention in recent years. It is estimated that more than 70% of cancers are arginine-auxotrophs due to the low expression of ASS1 24, and because treatment can be guided by the level of ASS1 expression, making it an attractive option for cancer therapy. Indeed, ADI-PEG20 which targets ASS1-low cancers has been widely used for this purpose 5, 6.

One of the challenges of ADI therapy is its development of resistance due to the restoration of ASS1 expression. As ASS1 is an essential enzyme for normal tissue to generate arginine, targeting ASS1 directly may cause unintended toxicity, which calls for the identification of additional targets contributing to resistance. In our study, overexpression of ASS1 in different types of arginine-auxotrophic cancer cells gave variable degrees of ADI resistance. Indeed, according to published literature, the mechanisms associated with ADI-resistance seem to vary. In melanoma cancer cells, Myc overexpression leads to ASS1 expression 17. In this study, we did not detect elevated Myc expression in either breast or prostate cancer ADI resistant cell lines. Thus, the path to the development of ADI resistance is likely to differ among different cancer types.

In an effort to further understand the mechanism(s) associated with ADI resistance in breast and prostate cancer cells and to identify potential targets to overcome resistance, we first developed ADI-resistant clones by step-up selection in the presence of ADI. Several clones were independently selected. While their individual transcriptomes are different, ASS1 upregulation is common among them, suggesting that ASS1 is necessary to confer resistance. The IC_50_ for ADI among these clones varies but is higher than 4.8 μg/mL in all of them. These resistant cells grow more slowly in the presence of ADI (Figure 1F), but are not dying (Figure 1G). The slow growth may account for the somewhat reduced cell viability in the absence of external arginine (Figure 1A). For comparative analyses, we also overexpressed ASS1 in the parental M231 clone to develop ADI resistant clones. Interestingly, they appear to be less resistant (IC_50_ = 0.18 μg/mL) than ADIR clones although having comparable level of ASS1 expression. The differential resistance to ADI were also demonstrated in xenograft models. These results prompted us to identify the additional resistant factors. We employed both genetic (CRISPR) and transcriptomic (RNA-seq) approaches, where both pinpoint the TREM1 pathway to be an important contributor to ADI resistance.

Inflammatory pathways including TREM1 were found to be upregulated in all the ADI-selected clones but not in ASS1-overexpressing clones. Knocking down or CRISPR knock-out of TREM1 diminishes ADI resistance of these clones. TREM1 pathway related genes such as CCL2, PLCg, NLR are upregulated, and phosphorylation signals associated with TREM1 are also activated.

CRISPR sg-library offers an unbiased screen to identify genes whose depletion can overcome resistance, and thus are targets for ADI resistance. TREM1 and CCL2 were both identified through this strategy, further supporting our hypothesis. In this screen, we did not detect ASS1 as a target, likely due to the requirement of ASS1 for M231 survival even in the absence of ADI treatment. Consistent with this notion, we were able to obtain knockdown but not knockout clones. In addition to TREM1 and CCL2, our screening also uncovered GNMT and GATM, enzymes involved in arginine metabolism to creatine. Their roles in ADI resistance require further investigation. It should be noted that in our survey of five prostate (PC3, CWR22Rv1, DU145) and breast cancer (MDA-MB-231 and BT-459) ADI-resistant cell lines, all restored ASS1 expression. In four of the five resistant cell lines, TREM or CCL2 expression is elevated, suggesting TREM1/CCL2 axis upregulation represents a common occurrence in ADI resistance. On the other hand, the fact that DU145 achieved full ADI resistance without the upregulation of TREM1 or CCL2 argues for additional resistant pathways. GNMT and GATM described above or c-Myc and Axl reported by Tsai *et al.* in melanoma 41 are likely additional candidates involved in ADI resistance. Although limited availability of ADI-resistant samples from clinical trials at present does not allow us to identify clinical correlates, our study nevertheless demonstrates the potential of the TREM1 pathway to confer resistance, and its inhibition may be considered as a strategy to overcome ADI resistance.

TREM1 is a cell surface receptor predominantly expressed in myeloid cells and functions to amplify inflammatory signals and enhance the release of cytokines and chemokines including CCL2 (MCP-1), IL-1α, IL-6, and TNFα 42, 43. In addition to infectious disease, recent evidence suggests TREM1 also plays a significant role in non-infectious diseases including cancer. TREM1 expression is elevated in many cancer types (TCGA) 44. While TREM1-associated neutrophils and macrophages contribute to the immune-escape and metastasis of tumors, aberrant expression of TREM1 is also found in tumor cells themselves as well as in established cell lines and provides growth, survival, and migration advantages 44. It has also been shown that in non-myeloid cells, TREM1 expression can be induced by nuclear receptor ligands 45.

In the present study, we found significantly elevated expression of TREM1 in independently selected ADI resistant clones from both breast and prostate cancer cell lines, suggesting a functional role of TREM1 in drug resistance. As these cells also overexpress ASS1, we asked if there could be a causal relationship between the expressions of TREM1 and ASS1. No consistent correlation was identified, indicating these upregulations are likely independent events. While knockdown or CRISPR-knockout of TREM1 renders resistant cells more sensitive to ADI, overexpression of TREM1 alone is not sufficient to confer full resistance to ADI (Figure S4G).

These data suggest that TREM1 alone cannot compensate for the loss of arginine, but contributes to robust survival or growth of resistant cells as an auxiliary factor to ASS1. This is particularly important for ASS1 overexpressor, as ASS1 has been shown to be a tumor suppressor that suppresses AKT signals 20 and diverts aspartate to arginine synthesis thus diminishing the nucleotide pool and inhibiting cell growth 3. We previously showed that overexpression of ASS1 indeed retards the growth of recipient cells 23, 46. TREM1 thus provides signals to allow ASS1 overexpressor to regain growth ability.

As to the question of how TREM1 contributes to growth and migration of ADIR, we found that as with myeloid cells, Syk and Btk, which are known to be expressed in breast cancer cells 47-49, are activated in ADIR clones, as are the downstream ERK and PI3K/AKT/mTOR and STAT3 signals 35, 50. We do not know the ligands which activate TREM1 in ADIR clones, but HMGA1 51-54, Peptidoglycan recognition receptor 1 (PGLYRP1) 35, 55, and eCIRP 56 are potential candidates. In M231-ADIR, PGLYRP1 and eCIRP were not detected, but HMGA1 was expressed and remains a possible candidate; its expression, however, is not elevated in resistant clones.

In strong support of TREM1 activation in ADIR is the upregulation of its well-known target chemokine CCL2. CCL2 has a strong link to chemotherapy and radiotherapy resistance and tumor progression in breast 39, 40, 57, prostate 38, 58-60, pancreatic 61, melanoma 62, lung 63, renal 64 and ovarian 65 cancers. Downstream signals of CCL2 such as ERK and PI3K/AKT provide a feed-forward loop to enforce TREM1 oncogenic effects 66. We showed that CCL2 contributes to ADIR's robust resistance and that knockdown or CRISPR-knockout of CCL2 reverses this phenotype.

In summary, the present study is designed to advance our understanding of resistance mechanisms to ADI-therapy and to uncover potential targets to overcome such resistance. We confirm that ASS1 expression is a necessary factor that presumably restores arginine metabolism; however, for full-resistance, TREM1/CCL2 axis appears to provide survival and growth functions in the cell models we studied. The latter could be considered as targets for overcoming ADI resistance.

## Supplementary Material

Supplementary figures and tables.

## Abbreviations

ADI: ADI-PEG20, pegylated arginine deiminase
ADIR: ADI resistance
ASS1: argininosuccinate synthetase 1
Cas9: CRISPR associated protein 9
CCL2: MCP-1, monocyte chemoattractant protein 1
CRISPR: clustered regularly interspaced short palindromic repeats
ELISA: Enzyme-linked immunosorbent assay
GSEA: Gene Set Enrichment Analysis
IC_50_: half maximal inhibitory concentration
IHC: Immunohistochemistry
IPA: Ingenuity Pathway Analysis
MFS: metastasis-free survival
MTT: 3-(4,5-Dimethylthiazol-2-yl)-2,5-diphenyltetrazolium bromide
NGS: next generation sequencing
OS: overall survival
PI: Propidium iodide
RFS: relapse-free survival
RNA-seq: RNA Sequencing
RT-PCR: real-time reverse transcription-quantitative PCR
RTCA: real-time cell analysis
sgRNA: single-guide RNA
shRNA: short hairpin RNA
TCGA: The Cancer Genome Atlas
TREM1: triggering receptor expressed on myeloid cells 1
