# Supplementary material for: Genome-wide CRISPR/Cas9 knockout screening uncovers a novel inflammatory pathway critical for resistance to arginine-deprivation therapy
Source: Theranostics. 2021 Jan 25;11(8):3624–41. doi: 10.7150/thno.51795 (PMC7914361; doi:10.7150/thno.51795)
Supplement: Supplementary file 1 — Supplementary figures and tables. [file thnov11p3624s1.pdf]

Figure S1

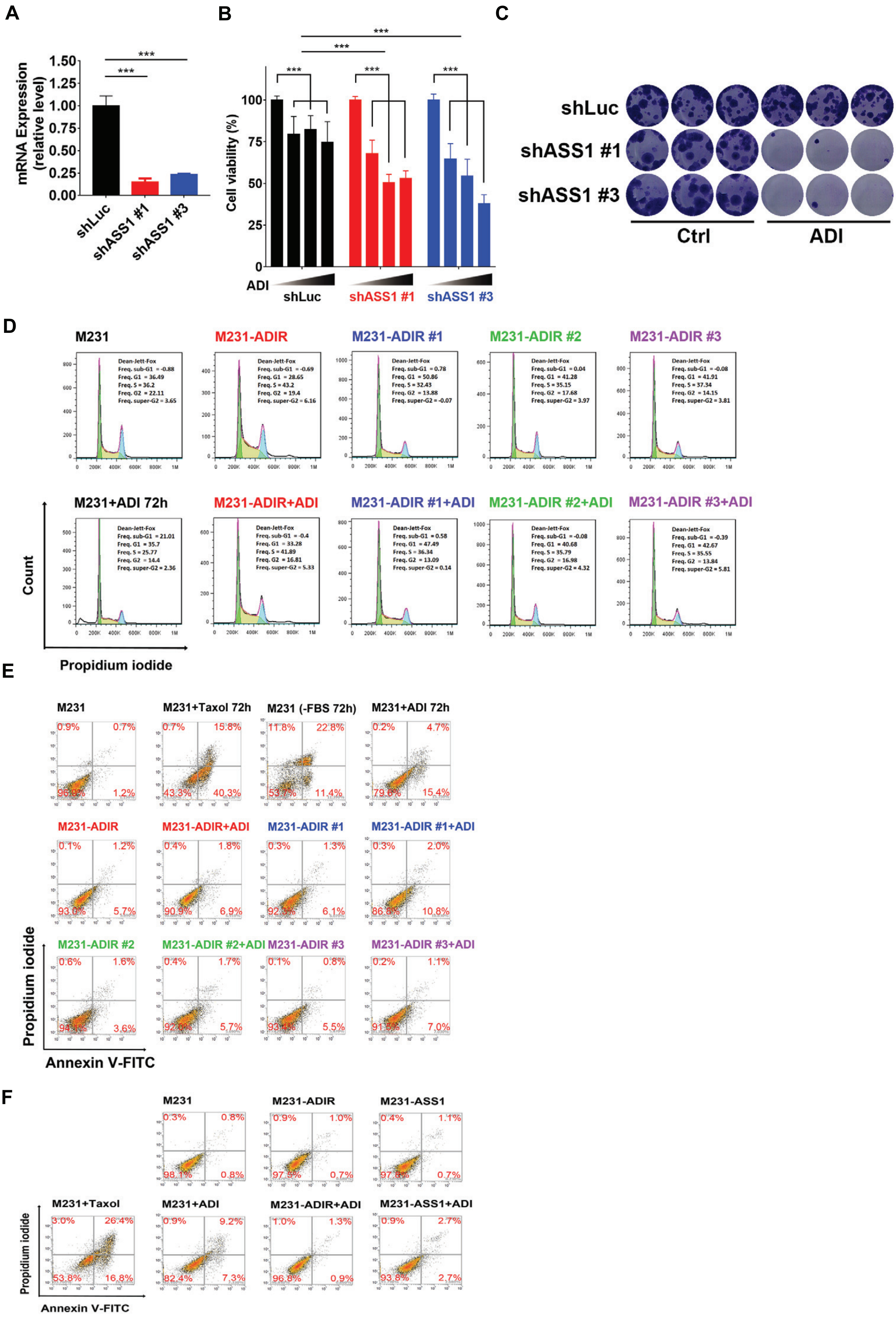

A
B
C
mRNA Expression (relative level)
1.50
1.25
1.00
0.75
0.50
0.25
0.00
***
shLuc
shASS1 #1
shASS1 #3
Cell viability (%)
100
75
50
25
0
ADI
shLuc
shASS1 #1
shASS1 #3
shLuc
shASS1 #1
shASS1 #3
Ctrl
ADI
D
M231
M231-ADIR
M231-ADIR #1
M231-ADIR #2
M231-ADIR #3
M231+ADI 72h
M231-ADIR+ADI
M231-ADIR #1+ADI
M231-ADIR #2+ADI
M231-ADIR #3+ADI
Count
Propidium iodide
E
M231
M231+Taxol 72h
M231 (-FBS 72h)
M231+ADI 72h
M231-ADIR
M231-ADIR+ADI
M231-ADIR #1
M231-ADIR #1+ADI
M231-ADIR #2
M231-ADIR #2+ADI
M231-ADIR #3
M231-ADIR #3+ADI
Propidium iodide
Annexin V-FITC
F
M231
M231-ADIR
M231-ASS1
M231+Taxol
M231+ADI
M231-ADIR+ADI
M231-ASS1+ADI
Propidium iodide
Annexin V-FITC

Figure S2

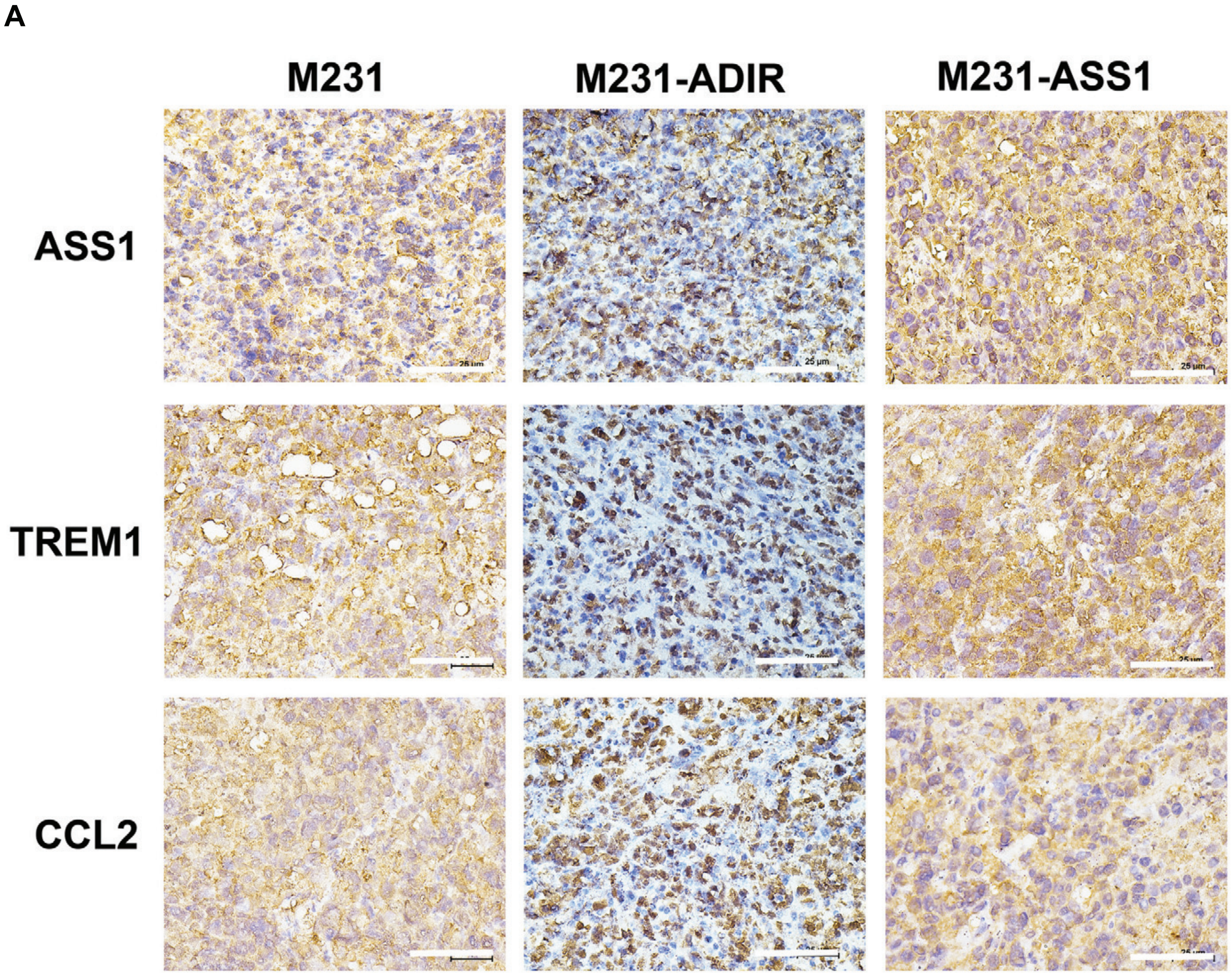
A
M231
M231-ADIR
M231-ASS1
ASS1
TREM1
CCL2
25 µm

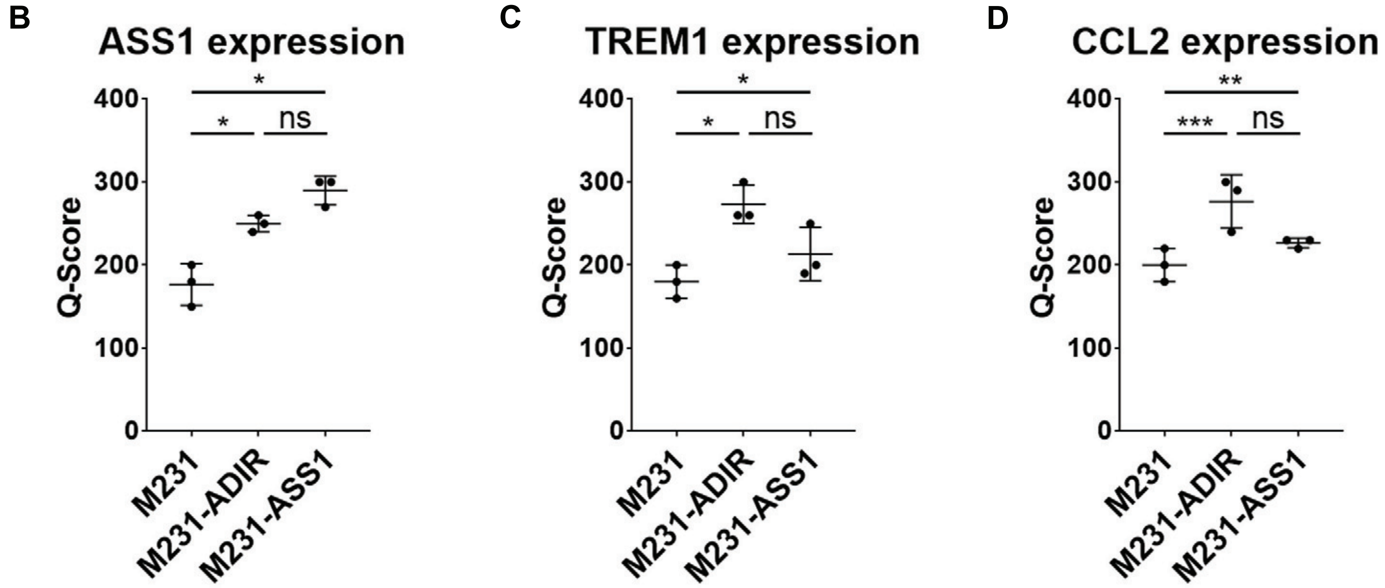
B
ASS1 expression
C
TREM1 expression
D
CCL2 expression
Q-Score
0
100
200
300
400
M231
M231-ADIR
M231-ASS1
*
ns
**
***

Figure S3

A

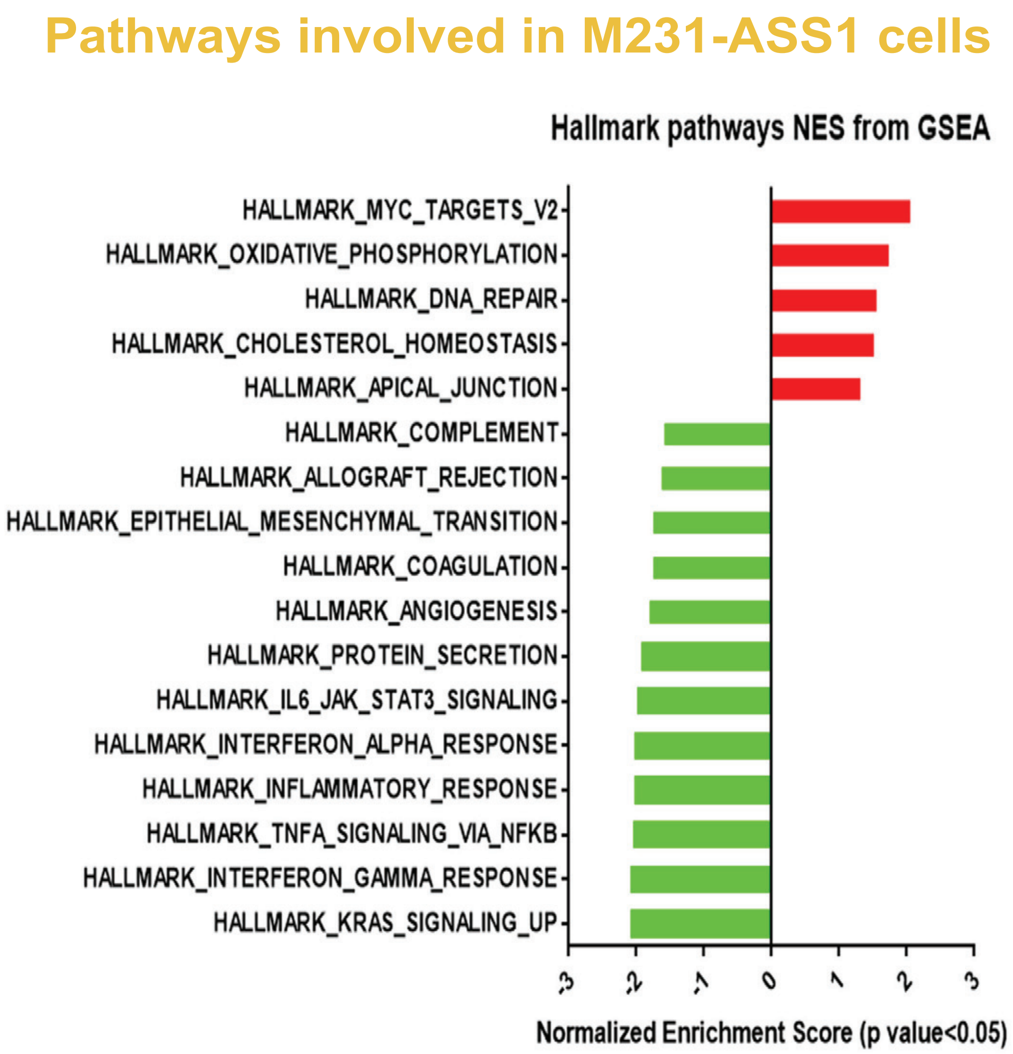

Pathways involved in M231-ASS1 cells
Hallmark pathways NES from GSEA
HALLMARK_MYC_TARGETS_V2
HALLMARK_OXIDATIVE_PHOSPHORYLATION
HALLMARK_DNA_REPAIR
HALLMARK_CHOLESTEROL_HOMEOSTASIS
HALLMARK_APICAL_JUNCTION
HALLMARK_COMPLEMENT
HALLMARK_ALLOGRAFT_REJECTION
HALLMARK_EPITHELIAL_MESENCHYMAL_TRANSITION
HALLMARK_COAGULATION
HALLMARK_ANGIOGENESIS
HALLMARK_PROTEIN_SECRETION
HALLMARK_IL6_JAK_STAT3_SIGNALING
HALLMARK_INTERFERON_ALPHA_RESPONSE
HALLMARK_INFLAMMATORY_RESPONSE
HALLMARK_TNFA_SIGNALING_VIA_NFKB
HALLMARK_INTERFERON_GAMMA_RESPONSE
HALLMARK_KRAS_SIGNALING_UP
-3
-2
-1
0
1
2
3
Normalized Enrichment Score (p value<0.05)

Figure S4

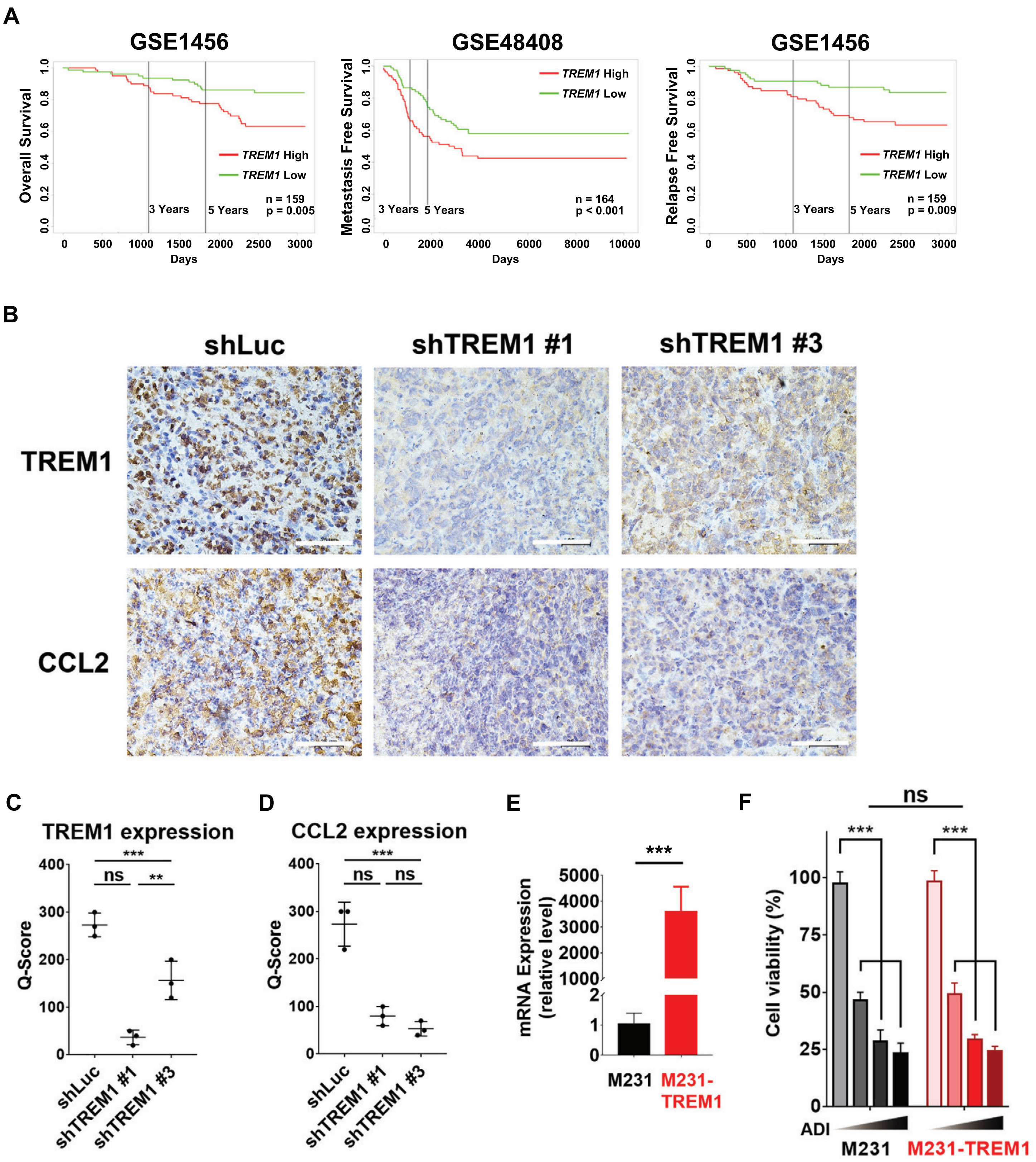
A
GSE1456
Overall Survival
TREM1 High
TREM1 Low
n = 159
p = 0.005
3 Years
5 Years
Days
GSE48408
Metastasis Free Survival
TREM1 High
TREM1 Low
n = 164
p < 0.001
3 Years
5 Years
Days
GSE1456
Relapse Free Survival
TREM1 High
TREM1 Low
n = 159
p = 0.009
3 Years
5 Years
Days
B
shLuc
shTREM1 #1
shTREM1 #3
TREM1
CCL2
C
TREM1 expression
Q-Score
ns
**
***
shLuc
shTREM1 #1
shTREM1 #3
D
CCL2 expression
Q-Score
ns
ns
***
shLuc
shTREM1 #1
shTREM1 #3
E
mRNA Expression (relative level)
***
M231
M231-TREM1
F
ns
***
***
Cell viability (%)
ADI
M231
M231-TREM1

Figure S5

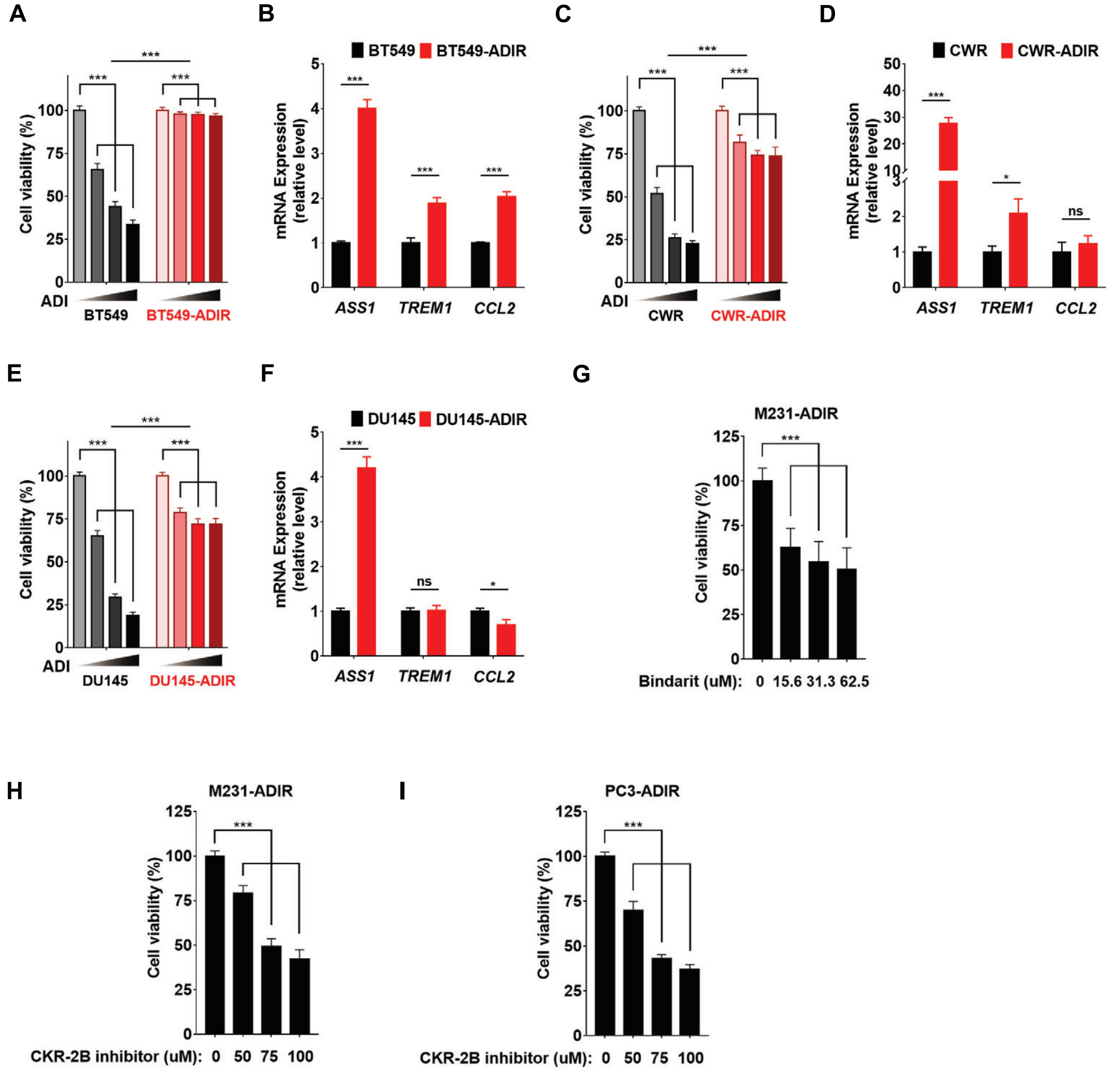
A
B
C
D
E
F
G
H
I
Cell viability (%)
ADI
BT549
BT549-ADIR
mRNA Expression (relative level)
ASS1
TREM1
CCL2
CWR
CWR-ADIR
DU145
DU145-ADIR
ns
M231-ADIR
Bindarit (uM): 0 15.6 31.3 62.5
CKR-2B inhibitor (uM): 0 50 75 100
PC3-ADIR

Figure S6

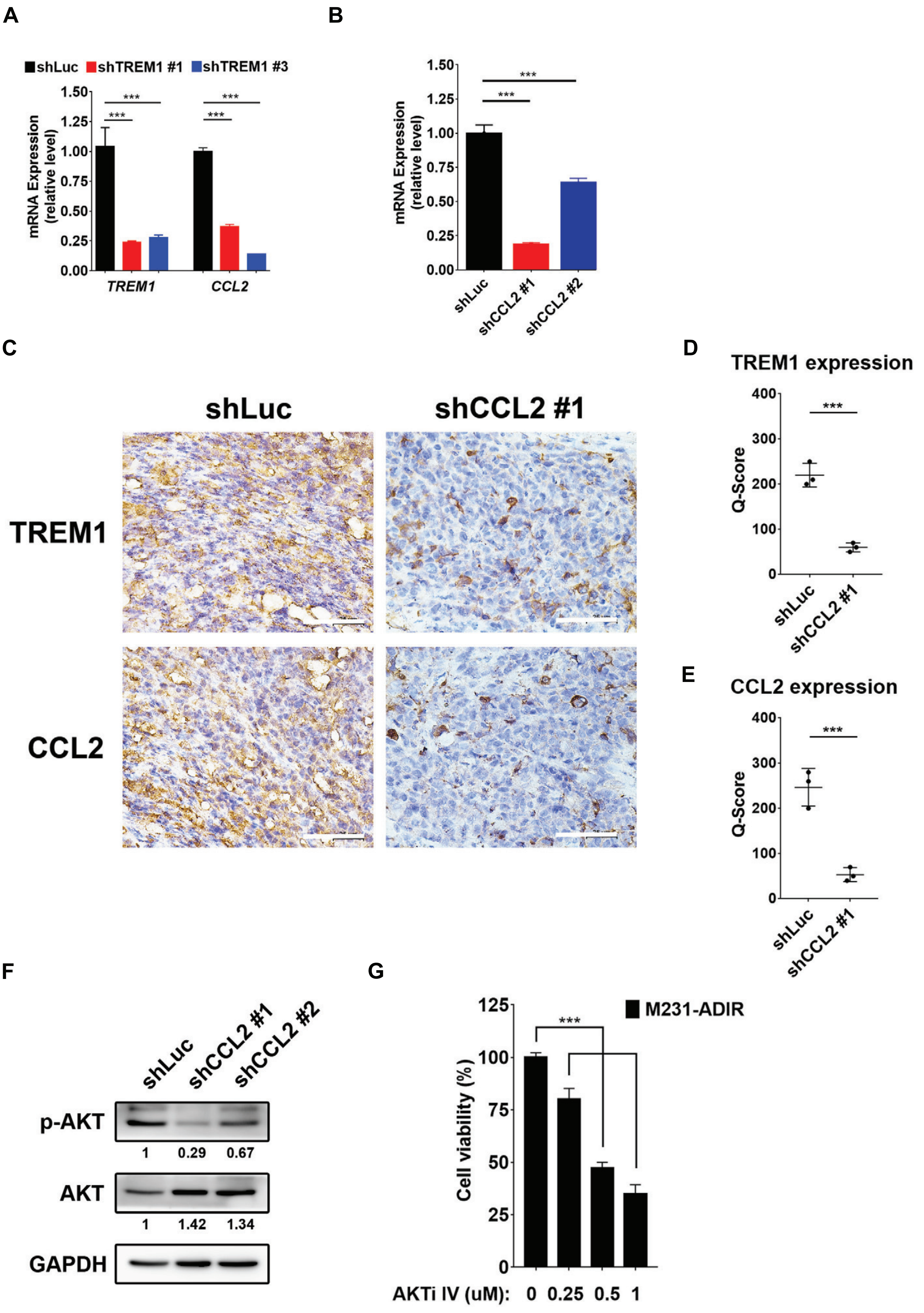
A
shLuc
shTREM1 #1
shTREM1 #3
mRNA Expression (relative level)
1.50
1.25
1.00
0.75
0.50
0.25
0.00
***
TREM1
CCL2
B
mRNA Expression (relative level)
shLuc
shCCL2 #1
shCCL2 #2
C
shLuc
shCCL2 #1
TREM1
CCL2
D
TREM1 expression
Q-Score
400
300
200
100
0
E
CCL2 expression
F
p-AKT
1 0.29 0.67
AKT
1 1.42 1.34
GAPDH
G
M231-ADIR
Cell viability (%)
125
100
75
50
25
0
AKTi IV (uM): 0 0.25 0.5 1

Figure S7

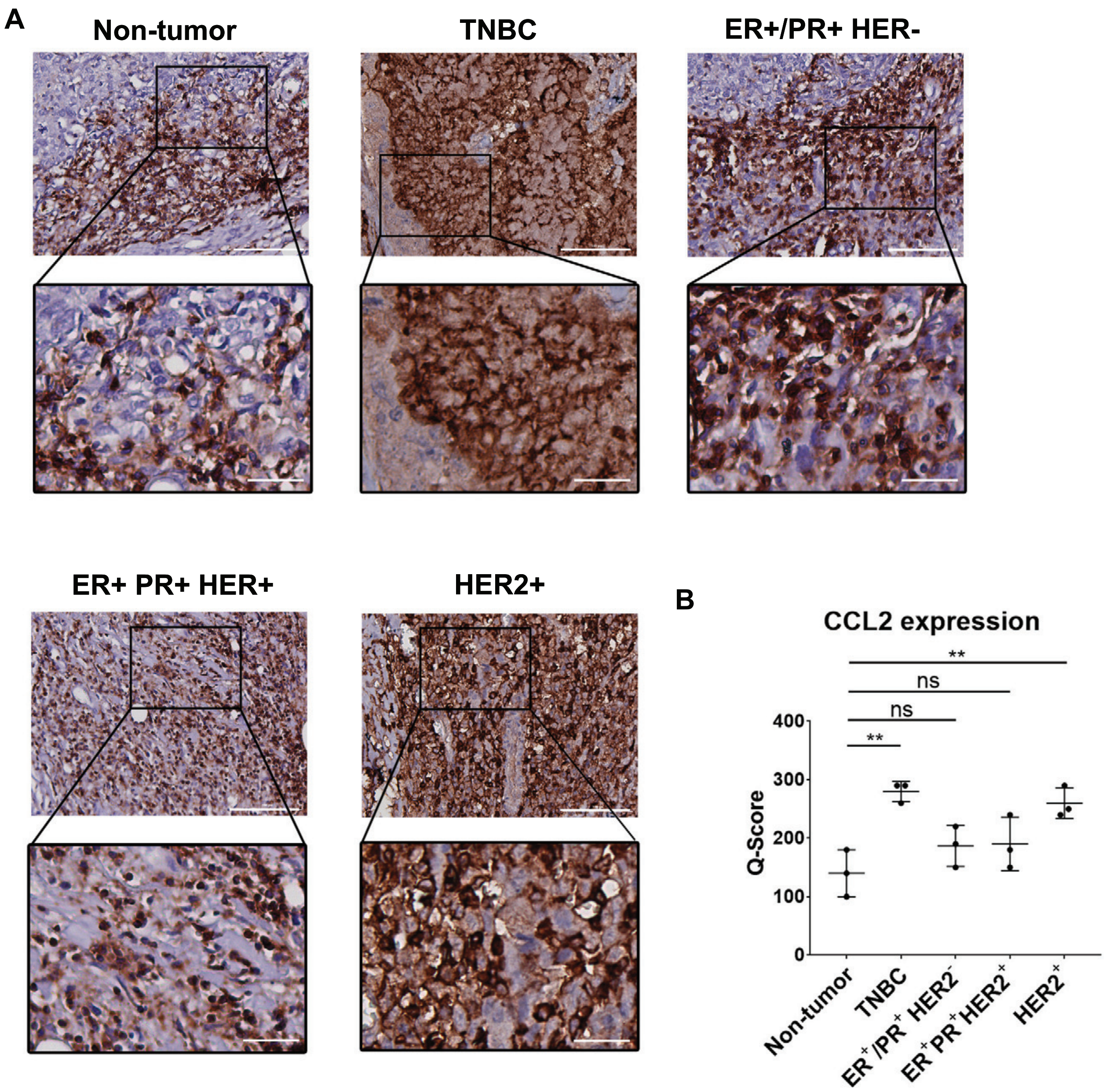
A
Non-tumor
TNBC
ER+/PR+ HER-
ER+ PR+ HER+
HER2+
B
CCL2 expression
**
ns
ns
**
400
300
200
100
0
Q-Score
Non-tumor
TNBC
ER+/PR+ HER2-
ER+PR+HER2+
HER2+

Figure S8

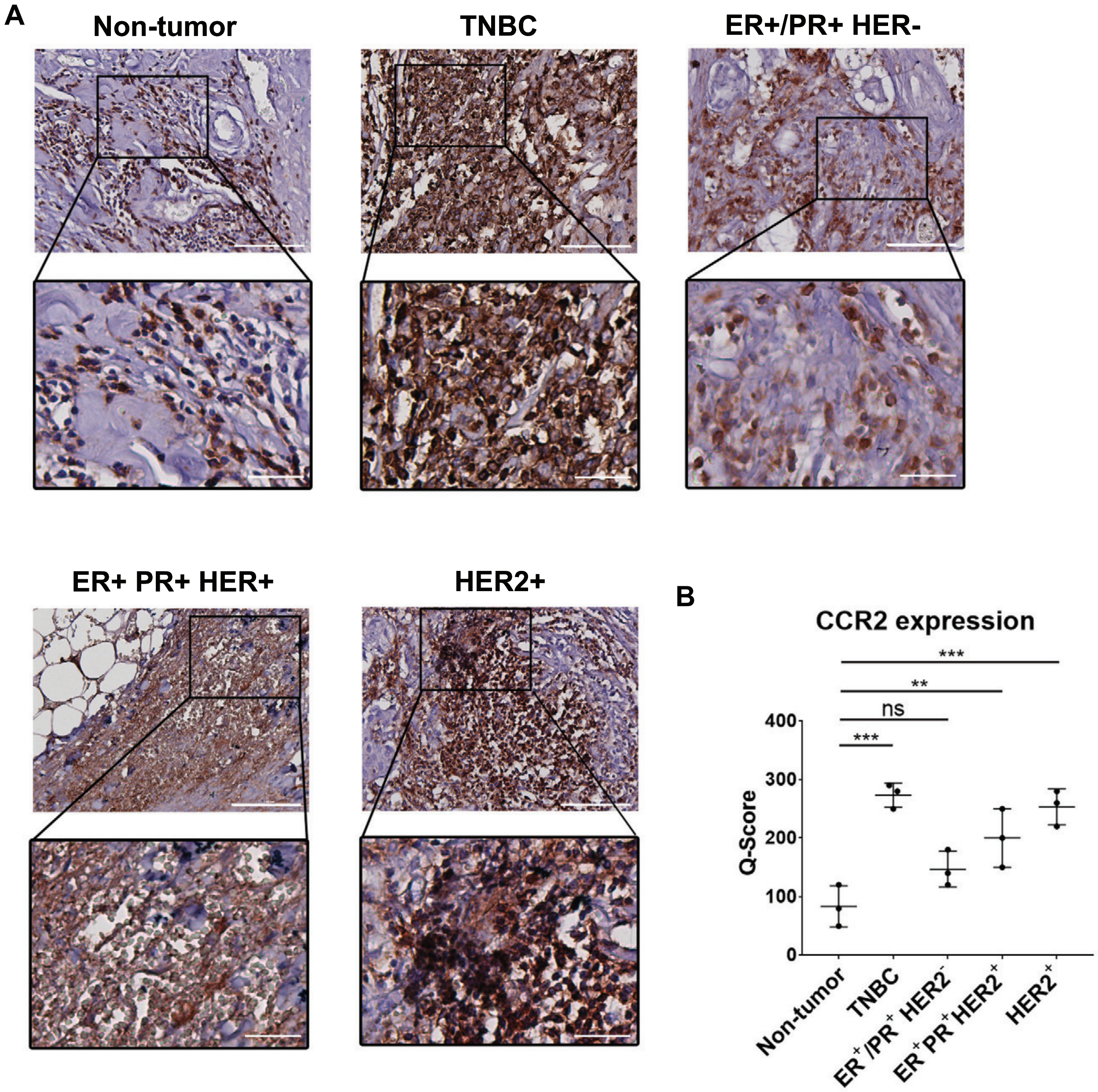
A
Non-tumor
TNBC
ER+/PR+ HER-
ER+ PR+ HER+
HER2+
B
CCR2 expression
***
**
ns
***
Q-Score
400
300
200
100
0
Non-tumor
TNBC
ER+/PR+ HER2-
ER+PR+HER2+
HER2+

Figure S9

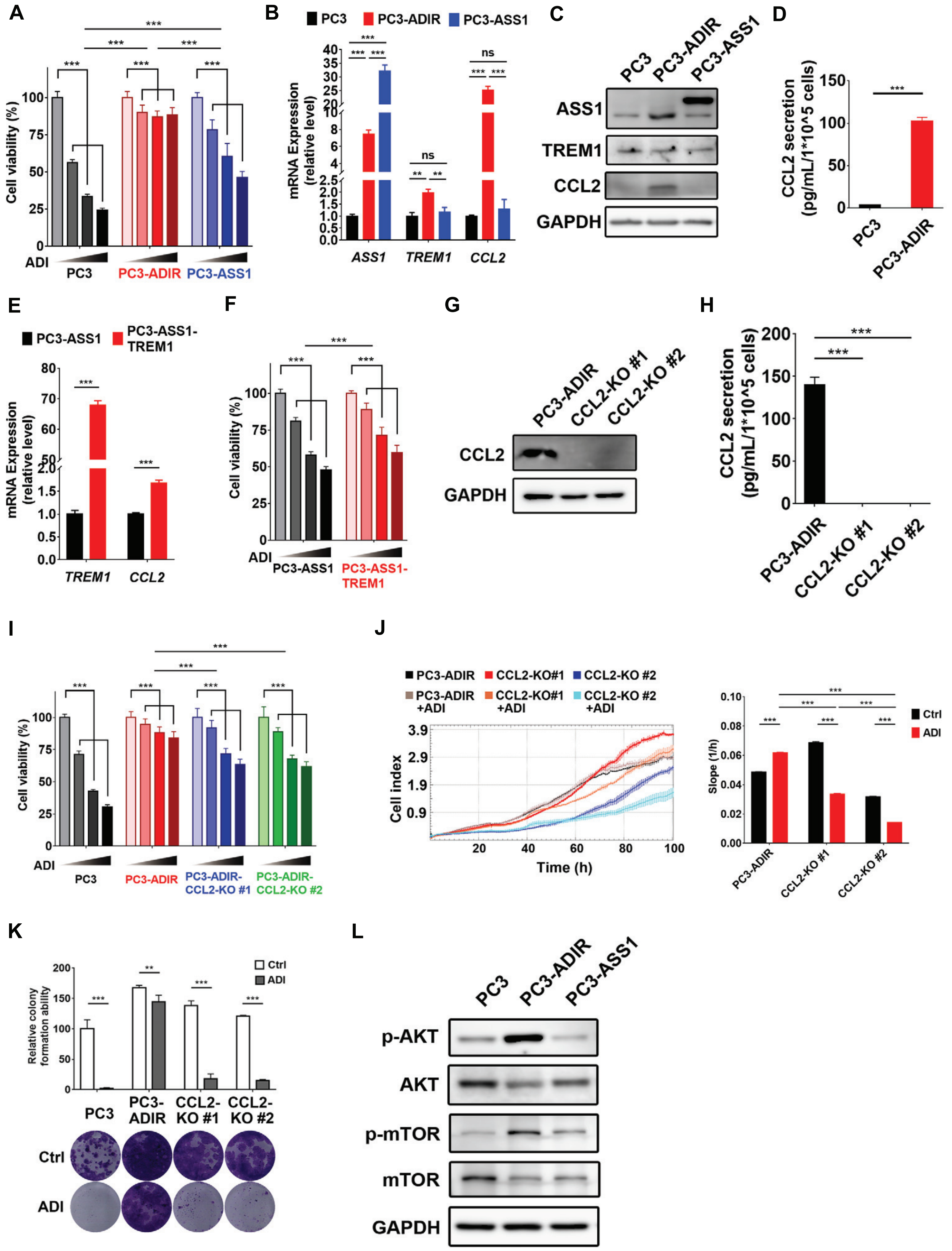
A
B
C
D
E
F
G
H
I
J
K
L
Cell viability (%)
ADI
PC3
PC3-ADIR
PC3-ASS1
mRNA Expression (relative level)
ASS1
TREM1
CCL2
GAPDH
CCL2 secretion (pg/mL/1*10^5 cells)
PC3-ASS1-TREM1
CCL2-KO #1
CCL2-KO #2
PC3-ADIR-CCL2-KO #1
PC3-ADIR-CCL2-KO #2
Cell index
Time (h)
Slope (1/h)
Ctrl
Relative colony formation ability
p-AKT
AKT
p-mTOR
mTOR

Table S1. Primers used for real-time PCR

Gene	QPCR primer sequence
RPLP0	Forward sequence 5’-TGG TCA TCC AGC AGG TGT TCG A-3’
	Reverse sequence 5’-ACA GAC ACT GGC AAC ATT GCG G-3’
ASS1	Forward sequence 5’-GAG GAT GCC TGA ATT CTA CA-3’
	Reverse sequence 5’-GTT GGT CAC CTT CAC AGG-3’
CCL2	Forward sequence 5’-AGA ATC ACC AGC AGC AAG TGT CC-3’
	Reverse sequence 5’-TCC TGA ACC CAC TTC TGC TTG G-3’
TREM1	Forward sequence 5’-CGA TGT CTC CAC TCC TGA CTC T-3’
	Reverse sequence 5’-CAG CAA ACA GGA CAG AGA AGA CC-3’

Table S2. shRNA clones used in this study

TRC clone	Clone ID	Target sequence
pLKO-shTREM1 #1	TRCN0000056753	CCGGTGTTCAACATTGTCATT
pLKO-shTREM1 #3	TRCN0000373808	GGCAGACCCTGGATGTGAAAT
pLKO-shASS1 #1	TRCN0000440576	CCCAAGTACAGGCGCTAATTG
pLKO-shASS1 #2	TRCN0000443685	ACGCAAAGCAACACGGGATTC
pLKO-shCCL2 #1	TRCN0000006279	GATGTGAAACATTATGCCTTA
pLKO-shCCL2 #2	TRCN0000006280	GCTGTTATAACTTCACCAATA
pLKO-shAKT1	TRCN0000288786	GATCCTCAAGAAGGAAGTCAT
pLKO-shSTAT3 #1	TRCN0000020843	GCAAAGAATCACATGCCACTT
pLKO-shSTAT3 #2	TRCN0000020842	GCACAATCTACGAAGAATCAA

Table S3. Antibodies used for western blotting

Antibody	Catalog number	Manufacturer
Phospho-Stat3 (Tyr705)	9145	Cell Signaling Technology
Stat3	4904	Cell Signaling Technology
Phospho-AKT (Ser473)	9271	Cell Signaling Technology
AKT	9272	Cell Signaling Technology
Phospho-p44/42 MAPK (Erk1/2) (Thr202/Tyr204)	4370	Cell Signaling Technology
p44/42 MAPK (Erk1/2)	9102	Cell Signaling Technology
Phospho-MTOR (Ser2448)	2971	Cell Signaling Technology
MTOR	2972	Cell Signaling Technology
Cleaved Caspase-3 (Asp175)	9664	Cell Signaling Technology
Caspase-3	9662	Cell Signaling Technology
ASS1	ab77590	Abcam
CCL2	ab214819	Abcam
TREM1	314902	Biolegend
